# Low‐Complexity Phase–Transition‐Based VO_2_ Transmissive Reconfigurable Intelligent Surface for Indoor Millimeter‐Wave Communications

**DOI:** 10.1002/nap2.70291

**Published:** 2026-09-08

**Authors:** Junghyeon Kim, Sumin Bark, Minjae Lee, Sungjoon Lim

**Affiliations:** ^1^ Department of Intelligent Semiconductor Engineering Chung‐Ang University Seoul Republic of Korea; ^2^ School of Electrical and Electronic Engineering Chung‐Ang University Seoul Republic of Korea

**Keywords:** low complexity, low cost, metasurface, reconfigurable intelligent surface, VO_2_

## Abstract

This paper presents a low‐complexity and low‐cost transmissive reconfigurable intelligent surface (TRIS) based on the phase‐change material vanadium dioxide (VO_2_) for indoor millimeter‐wave (mm‐wave) communications. Although mm‐wave systems enable ultra‐high data rates and low latency, their performance is limited by severe path loss and non‐line‐of‐sight propagation. RISs offer a promising solution by dynamically controlling electromagnetic wave propagation. In particular, TRISs provide greater deployment flexibility than reflective RISs in indoor environments where transmitter–receiver links are blocked by walls, windows, or other barriers. However, wide phase tunability in TRISs typically requires complex circuit architectures. Moreover, conventional active components such as PIN and varactor diodes suffer from significant parasitic effects at mm‐wave frequencies, which degrade performance and increase design complexity. To address these limitations, the proposed TRIS exploits the metal–insulator phase transition of VO_2_ and geometrically engineered VO_2_ switch patterns to achieve flexible phase reconfiguration without complex biasing networks or lossy lumped components. The design is validated through theoretical analysis, full‐wave simulations, and measurements. The prototype steers transmitted waves from 0° to ± 20° at 65 GHz and achieves up to approximately 15 dB transmission enhancement between the programmed and reference states across 64–66 GHz, demonstrating its potential for indoor mm‐wave and WiGig applications.

## Graphical abstract

We demonstrate a VO_2_‐based transmissive reconfigurable intelligent surface for indoor millimeter‐wave communication. The TRIS replaces conventional PIN and varactor diodes with geometrically tailored VO_2_ switches, enabling transmitted phase control through phase‐transition‐based switching. The fabricated TRIS enables VO_2_ switch‐based beam steering, offering a low‐cost and low‐complexity hardware platform with potential for indoor millimeter‐wave beam‐control applications.

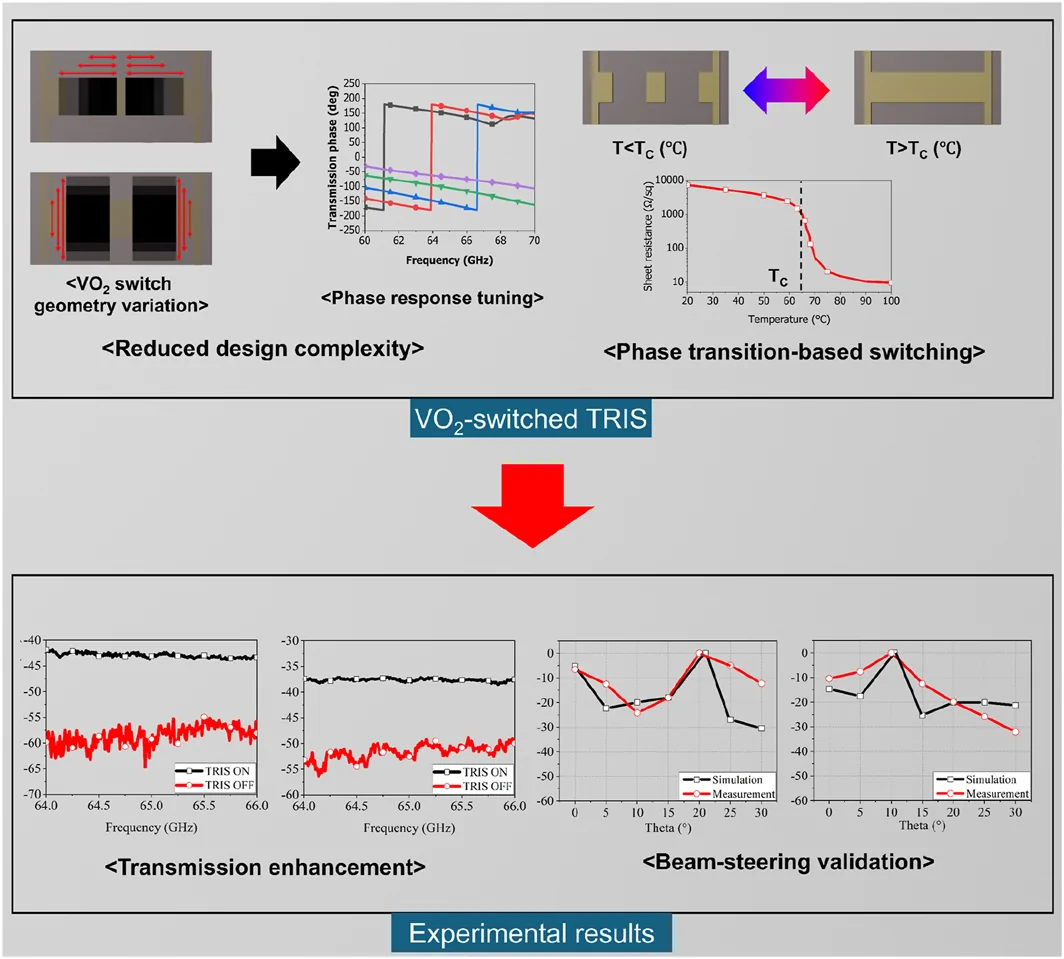

## 1 Introduction

With the widespread adoption of fifth‐generation wireless communication systems, increasing attention has been paid to high‐frequency and high‐performance electronic components for advanced communication platforms [1, 2, 3]. Millimeter‐wave (mm‐wave) communication, characterized by ultra‐high speed, high connectivity, and ultra‐low latency, is expected to broaden the scope of various radio‐frequency (RF) applications including augmented reality, virtual reality, autonomous driving, and holography [4, 5, 6]. However, its operation in high‐frequency bands makes it susceptible to considerable path loss and pronounced directionality, resulting in link failures under non‐line‐of‐sight conditions. These limitations hinder the commercialization of mm‐wave communication, prompting the exploration of several solutions including low‐Earth orbit (LEO) satellite communication systems [7, 8], unmanned aerial vehicle (UAV) communication frameworks [9, 10], beamforming antennas [11], and reconfigurable intelligent surfaces (RISs). However, each of these approaches also presents inherent limitations. For instance, LEO satellite communication necessitates high‐gain active antennas, whereas limited satellite dimensions introduce considerable antenna design complexity [12]. Further, UAV communication is subject to strict size, weight and power constraints and experiences issues such as limited coverage and vibration‐induced signal fading [13]. Moreover, beamforming arrays are prone to blockage and encounter packaging, wiring, and power‐related challenges during integration [14]. Meanwhile, RISs are regarded as particularly promising owing to their low power consumption, reduced complexity, and low cost, demonstrating the potential to overcome the limitations of preceding technologies [15, 16, 17].

Notably, RISs are devices designed to reflect or transmit incident electromagnetic waves at specific angles, thereby mitigating path loss, and in particular, non‐line‐of‐sight communication failures [18, 19, 20]. These devices are generally classified as either reflective or transmissive, depending on their operating mode. In particular, reflective RISs extend indoor coverage by forming line‐of‐sight paths around obstacles and require the transmitter and receiver to be located in the same half‐space. In contrast, transmissive RISs (TRISs) enhance indoor coverage in environments where the transmitter and receiver are separated by obstacles such as walls or windows. Generally, because indoor environments contain multiple physical barriers, including walls, doors, and windows, TRISs offer greater deployment flexibility and coverage enhancement than their reflective counterparts [21]. However, TRISs are more difficult to design and implement than reflective RISs. To implement a 1‐bit RIS, low loss and a phase difference close to 180° between the two states must be achieved simultaneously. In reflective RISs, the reflection phase can be significantly varied relatively easily by utilizing a ground‐plane‐backed structure. In contrast, because TRISs cannot employ a ground plane, it is difficult for a single‐layer structure to simultaneously achieve low insertion loss and a 180° phase difference. To overcome these limitations, previous studies have utilized multilayer FSSs or receive–transmit structures, Huygens metasurfaces [22], and Pancharatnam–Berry phase‐based structures [23]. However, when these approaches are applied to high‐frequency bands, such as the mm‐wave band, they face additional design challenges, including tight manufacturing tolerances and increased parasitic losses.

In general, TRIS designs commonly employ RF switches, including PIN and varactor diodes, to achieve two or more reconfigurable states. Although these devices are favored in microwave‐band systems owing to their fast switching speeds, high reliability and low power consumption, their performance degrades notably beyond the mm‐wave range, limiting their applicability in high‐frequency TRISs. For instance, the Q‐factor of varactor diodes declines with increasing frequency [24, 25], whereas PIN diodes experience high insertion loss in the mm‐wave region owing to parasitic components introduced during packaging [26]. Following this trend, most diode‐based TRIS studies have been conducted in frequency bands below 40 GHz, whereas TRISs operating above 40 GHz have been relatively rarely reported. Although W‐band PIN diodes, such as the MACOM MASW‐011111‐DIE, are commercially available, their unit price of approximately $216 makes them impractical for large‐scale TRIS implementations. To achieve phase control in a TRIS, at least two tunable elements are typically required per unit cell. For example, a 20 × 20 TRIS array would require 800 RF switches, and the number of required RF switches increases as the array size increases. Consequently, implementing TRISs in the mm‐wave band remains challenging in terms of both performance and cost.

Despite these challenges, several researchers have investigated diode‐based TRISs operating at mm‐wave to sub‐terahertz frequencies. For instance, Pan et al. [27] developed a TRIS operating at 97 GHz, incorporating two PIN diodes per unit cell and a two‐layer dielectric structure. Although this surface achieved phase variation, it exhibited an insertion loss of approximately 11 dB, approximately 6 dB above the ideal, and was limited to a small 32 × 2 array. This result indicates that implementing PIN diodes at mm‐wave frequencies often leads to elevated insertion loss, primarily owing to parasitic effects at high frequencies. In another study, Shen et al. [23] fabricated a TRIS operating at 100 GHz using Schottky diodes. This surface design achieved a relatively low insertion loss of 2.8 dB, enabled phase variation through a two‐layer structure, and achieved beam scanning up to ± 60°. However, the prototype was limited to an 8 × 8 array, and the design was further constrained by printed circuit board (PCB) processing tolerances, suggesting that error accumulation could become severe when scaled to large apertures. Furthermore, using conductive adhesive for attaching Schottky diodes hinders large‐area fabrication. To overcome these limitations, this study proposes a low‐complexity low‐cost TRIS employing a screen‐printable vanadium dioxide (VO_2_) switch.

Notably, VO_2_ is a phase‐change material whose resistance decreases with increasing temperature, prompting a sharp phase transition at approximately 67°C. This property allows it to operate as an RF switch with ON and OFF states, analogous to a PIN diode. Although VO_2_ switches are slower than their PIN counterparts, reported switching speeds of around 0.4 µs [28] indicate that they can still support relatively fast reconfigurable operation. A key advantage of VO_2_ is its ability to be deposited as a thin film without using conductive epoxy or soldering, thereby reducing parasitic components. This characteristic has spurred increasing interest in VO_2_ for terahertz‐band applications, prompting a growing body of related research [29, 30]. VO_2_ switches are typically realized using thin‐film processes; however, high cost, wafer‐size limitations, and slow fabrication speeds have constrained their application in RF systems [28, 31]. Nevertheless, more recently, screen‐printed VO_2_ patterning techniques have enabled low‐cost large‐area fabrication, helping to mitigate these challenges [28]. Consequently, VO_2_ has been actively explored for frequency‐reconfigurable antennas [32, 33], filters [28, 34], and RISs. In particular, research on VO_2_‐based RISs has primarily focused on reflective designs operating at 28 and 100 GHz [31, 35]. Notably, reflective RISs can be realized using a single VO_2_ switch per unit cell in a single‐layer structure, enabling the straightforward integration of VO_2_ printing processes into existing designs. In contrast, the recently introduced TRIS employs a binary switching scheme that demands independent control of two states (e.g., 0/1 or 1/0) within the same plane [23]. However, in thermally operated VO_2_ switches, thermal diffusion affects adjacent elements, complicating the realization of such a dual‐state structure. Accordingly, a new strategy is required to implement a low‐cost TRIS using VO_2_ switches.

In response, herein, we developed a low‐complexity, low‐cost, and phase‐transition‐based VO_2_ TRIS. The TRIS unit cell was designed with a four‐layer FSS to mitigate the influence of thermal diffusion on the switching behavior. This was accomplished by structuring the system such that its state changed only when all VO_2_ switches turned ON or OFF simultaneously. Figure 1 presents a conceptual illustration of the proposed VO_2_‐based TRIS. The proposed VO_2_‐based TRIS offers two major advantages. First, by leveraging the geometric flexibility of the VO_2_ switch, the required phase shifts can be readily implemented over the entire target frequency band. In particular, such flexibility is difficult to achieve with RF switches such as PIN diodes or varactor diodes, because their fixed dimensions inherently complicate TRIS design. In contrast, the screen‐printed VO_2_ switches used in this work provide greater freedom in pattern design. This advantage simplifies the phase‐shifting mechanism of the TRIS, thereby substantially reducing the design complexity. Second, the use of screen‐printed VO_2_ switches offers a potentially cost‐effective route compared with conventional semiconductor‐based RF switches. As discussed earlier, increasing the number of TRIS arrays requires a corresponding increase in the number of tunable elements, which can substantially increase the fabrication cost. By employing VO_2_ switches, the proposed approach alleviates this cost burden in scalable TRIS implementation. The proposed VO_2_‐based TRIS was fabricated using PCB processes, screen printing, and 3D printing, and its performance was experimentally verified.

**FIGURE 1:**
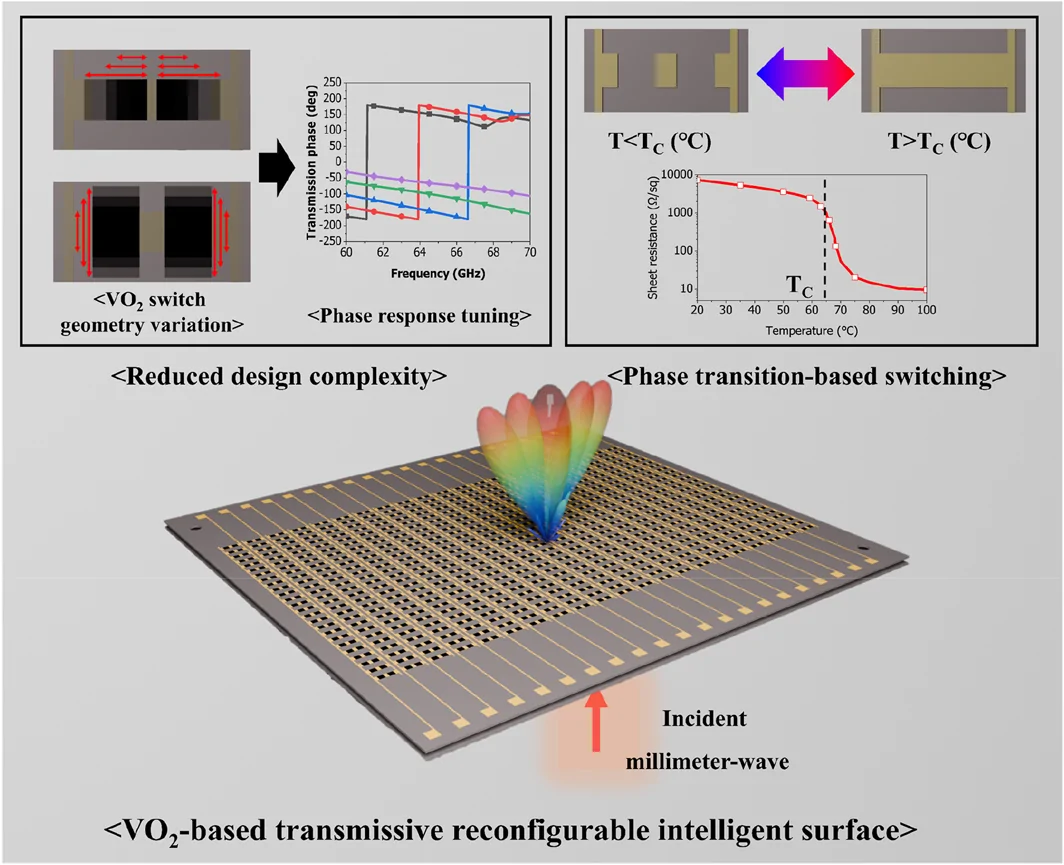
Conceptual illustration of the proposed low‐complexity, phase‐transition‐based VO_2_ TRIS.

## 2 Results

### 2.1 TRIS Unit Cell Design and Simulation

Figure 2a–c depict the bird's‐eye, top and bottom views of the proposed phase‐transition‐based VO_2_ TRIS unit cell, respectively. To fabricate the TRIS for mm‐wave‐band operation, the Rogers 5880 substrate, with a dielectric constant of 2.2 and a loss tangent of 0.0009, was employed. Further, to mitigate potential isolation degradation induced by heat diffusion during VO_2_ switching, a four‐layer FSS‐based unit cell was adopted. As illustrated in Figure 2b, this unit cell comprises four layers, and its ON and OFF states are realized through the simultaneous activation of VO_2_ switches. During analysis, the unit cell was simulated using Ansys HFSS under master–slave boundary conditions (Supporting Information S1: Note 1). Figure 2d compares the transmission coefficients of the proposed TRIS unit cell in the ideal ON and OFF states with those simulated using VO_2_ switches with sheet resistances of 10 Ω/sq and 12,000 Ω/sq. Notably, these sheet resistances (10 Ω/sq and 12,000 Ω/sq) were sourced from our previous measurements of a VO_2_ switch in the ON and OFF states [35]. Notably, for the VO_2_ switch with a sheet resistance of 10 Ω/sq, the transmission coefficient remains above −6 dB across 60–70 GHz. For the VO_2_ switch with a sheet resistance of 12,000 Ω/sq, the transmission coefficient remains above −6 dB from 60 to 68 GHz. The ON and OFF states display opposite trends until both reach −3.13 dB at 63.93 GHz, after which they diverge again. Relative to the ideal case, the ON state of the VO_2_ switch introduces approximately 2.5 dB of additional insertion loss, while the OFF state incurs an additional insertion loss of approximately 0.5 dB. This increase results from the higher effective resistance associated with the longer VO_2_ switch length ($v_{x}$). Although a longer VO_2_ switch raises insertion loss in the ON state, it promotes the desired phase‐shifting behavior. In our design, to achieve relatively uniform transmission coefficient between the ON and OFF states and ensure stable phase‐shifting behavior, two VO_2_ switches were employed per unit cell in one layer (Supporting Information S1: Note 2). Figure 2e presents the simulated transmission phase responses of the proposed TRIS unit cell incorporating VO_2_ switches with sheet resistances of 10 Ω/sq and 12,000 Ω/sq. The results indicate that the proposed TRIS unit cell achieves a phase variation of 180° ± 20° across 60–66.3 GHz. This behavior is readily achieved by adjusting the size of the VO_2_ switch, and the underlying mechanism is detailed in the section on phase variation with respect to VO_2_ switch dimensions.

**FIGURE 2:**
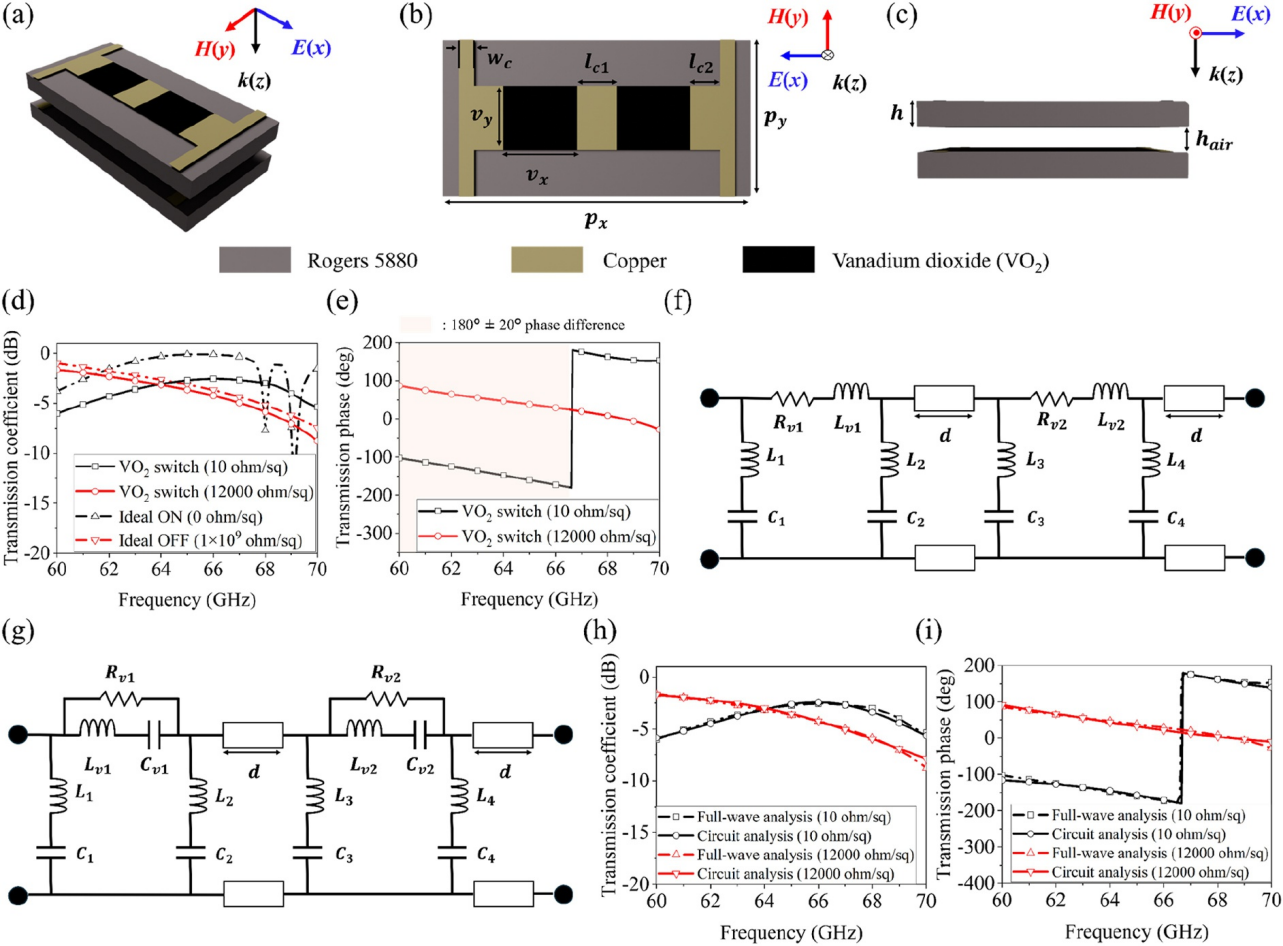
Unit cell structure of the proposed phase‐transition‐based VO_2_ TRIS and corresponding analysis results. (a–c) Bird's‐eye, top and side views of the unit cell ($p_{x}$ = 6.3 mm, $p_{y}$ = 3.2 mm, $w_{c}$ = 0.3 mm, $l_{c1}$ = 0.6 mm, $l_{c2}$ = 0.8 mm, $v_{x}$ = 1.5 mm, $v_{y}$ = 1.3 mm, h = 0.51 mm and $h_{\text{air}}$ = 0.5 mm). (d) Simulated transmission coefficients of the proposed TRIS unit cell in the ideal ON and OFF states, alongside those simulated using VO_2_ switches with sheet resistances of 10 Ω/sq and 12,000 Ω/sq. (e) Simulated transmission phases of the proposed TRIS unit cell in the ON and OFF states. (f, g) Equivalent circuits of the proposed TRIS with the VO_2_ switch in the ON and OFF states. (h, i) Comparison of transmission coefficients and transmission phase results obtained from full‐wave and circuit analyses for the VO_2_ switch in the ON and OFF states.

Next, we performed a circuit analysis of the proposed TRIS unit cell. Figure 2f presents the equivalent circuit model of the VO_2_ switch in the ON state. In this configuration, the parallel LC circuit represents the copper‐based meta‐pattern, whereas the series RL circuit corresponds to the VO_2_ switch. In the ON state, the VO_2_ switch acts as a conductor, rendering its capacitive effect negligible and allowing it to be approximated as a series RL circuit [35]. The two‐layer metamaterial pattern is thus modeled as two parallel LC circuits in parallel with a series RL branch. Here, *d* denotes the thickness of the Rogers 5880 substrate. For the ON state of the VO_2_ switch, the circuit parameters are as follows: $L_{1}$ = 7 nH, $L_{2}$ = 0.64 nH, $L_{3}$ = 10.5 nH, $L_{4}$ = 0.79 nH, $L_{v1}$ = 3.52 nH, $L_{v2}$ = 7.2 nH, $C_{1}$ = 4 fF, $C_{2}$ = 2.19 fF, $C_{3}$ = 20 pF, $C_{4}$ = 1.88 fF, $R_{v1}$ = 27 Ω, and $R_{v2}$ = 27 Ω. Figure 2g illustrates the equivalent circuit model of the VO_2_ switch in the OFF state. Although the VO_2_ switch is modeled as a series RL circuit in the ON state, it is represented by a series LC circuit with a parallel resistance in the OFF state. This configuration reflects the fact that the VO_2_ switch does not exhibit infinite resistance when OFF, meaning that the inductive component cannot be entirely neglected. Consequently, the resistive component must also be included. For the OFF state of the VO_2_ switch, the circuit parameters are as follows: $L_{1}$ = 1.3 nH, $L_{2}$ = 0.016 nH, $L_{3}$ = 0.124 nH, $L_{4}$ = 0.32 nH, $L_{v1}$ = 0.001 nH, $L_{v2}$ = 0.002 nH, $C_{1}$ = 8.3 fF, $C_{2}$ = 0.113 pF, $C_{3}$ = 0.106 pF, $C_{4}$ = 2.31 pF, $C_{v1}$ = 0.03 pF, $C_{v2}$ = 0.025 pF, $R_{v1}$ = 3500 Ω, and $R_{v2}$ = 3500 Ω. We used the Keysight Advanced Design System for circuit analysis and compared the simulated responses with the corresponding full‐wave analysis results. Figure 2h,i compare the transmission coefficients and phases derived from circuit and full‐wave analyses. In both the ON and OFF states of the VO_2_ switch, the results are in close agreement across the two methods. This consistency confirms the accuracy of the proposed equivalent circuit models for the VO_2_‐based TRIS unit cell.

We additionally investigated the angular stability of the proposed VO_2_‐based TRIS unit cell under oblique incidence. Figure 3a,b show the simulated transmission coefficients in the OFF and ON states, respectively. In the OFF state, an additional resonance appears above 66 GHz for incidence angles greater than 20°, whereas the transmission magnitude generally increases with incidence angle. In contrast, the transmission magnitude in the ON state gradually decreases with increasing incidence angle and falls below −10 dB at 60°. Smith‐chart analysis indicates that these opposite trends are associated with the angle‐dependent variation in the effective impedance of the unit cell (Supporting Information S1: Note 3). Nevertheless, the transmission coefficient remains above −6 dB for incidence angles up to 40° over the target 63–66 GHz band.

**FIGURE 3:**
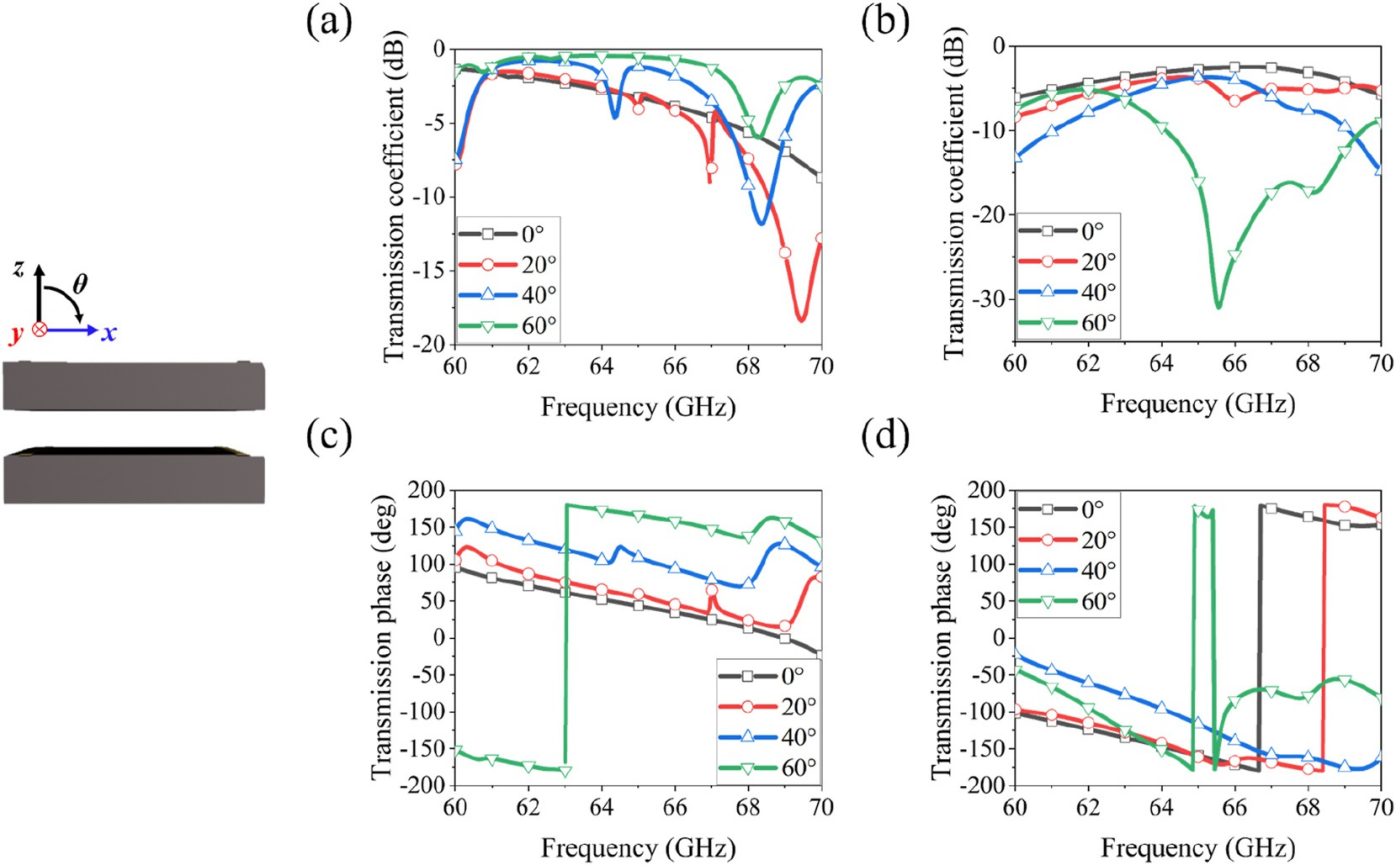
Simulated unit‐cell responses under oblique incidence for an *x*‐polarized wave: (a) transmission coefficient in the OFF state, (b) transmission coefficient in the ON state, (c) transmission phase in the OFF state, and (d) transmission phase in the ON state.

Figure 3c,d show the corresponding transmission‐phase responses. At 64 GHz, the OFF‐state phase changes from 52° at normal incidence to 173° at 60°, whereas the ON‐state phase exhibits a smaller variation up to 20° followed by a larger change at higher incidence angles. Importantly, the phase difference between the ON and OFF states remains within 180° ± 20° up to an incidence angle of 40° over the operating band but deviates from this range at 60°. These results demonstrate that the proposed unit cell maintains its intended 1‐bit transmission characteristics for oblique incidence up to approximately 40°, whereas its performance degrades at larger incidence angles.

### 2.2 Design Process of the Proposed TRIS Unit Cell

Figure 4a depicts the staged design process of the proposed TRIS unit cell. The process began with an initial structure wherein the VO_2_ switch was comparable in size to a diode. To induce phase variation, the structure was modified into a four‐layer configuration, and the unit‐cell width ($p_{x}$) was increased to provide sufficient phase shift while maintaining a high transmission coefficient in both the ON and OFF states. Finally, increasing the number of VO_2_ switches per layer from one to two further improved the transmission coefficient and stabilized the phase response. Figure 4b displays the evolution of the transmission coefficient across the design stages for the VO_2_ switch in the ON state. In Step 1, the transmission coefficient remains below −4 dB across the entire 60–70 GHz band. Further, in Step 2, converting the structure into a multilayer configuration further reduces the transmission coefficient. Enlarging the VO_2_ switch yields a transmission coefficient of approximately −6.5 dB or lower. The proposed TRIS unit cell achieves an improvement of around 0.5 dB over the Step 3 design. Figure 4c illustrates the transmission coefficient at each design stage when the VO_2_ switch is in the OFF state. In Step 1, unlike the ON state, the structure yields a low transmission coefficient of −5 dB or less across the entire frequency band. In Step 2, the structure is modified into a multilayer configuration, and an increase in the transmission coefficient is observed near 61 GHz. The structure corresponding to Step 3 exhibits a greater transmission coefficient than those corresponding to Steps 1 and 2 across most of the frequency range, except at the 61 GHz resonance. Adding a second VO_2_ switch to each layer reduces the transmission coefficient again. However, both the ON and OFF states must be considered in TRIS design. As depicted in Figure 4b, the split‐switch configuration improves the ON‐state transmission coefficient compared to the Step 3 design. This modification thus supports balanced optimization of the overall TRIS response. Figure 4d illustrates the simulated transmission phase difference between the ON and OFF states of the VO_2_ switch at each design stage. In Step 1, the phase difference is limited to approximately 60°. To increase this, the design was modified into a multilayer structure. In Step 2, a phase difference of approximately 200° was observed in specific frequency bands, but both the phase difference and the transmission coefficient remained suboptimal. In Step 3, enlarging the VO_2_ switch yielded a phase difference of around 240°. To reduce this, the switch was split into two parts. With further optimization, the proposed design achieved a phase‐variation bandwidth that satisfies the required performance criteria across the 60–66.3 GHz range. Notably, adjusting the VO_2_ switch size enabled phase‐difference optimization while reducing TRIS design complexity, where such optimization is typically difficult.

**FIGURE 4:**
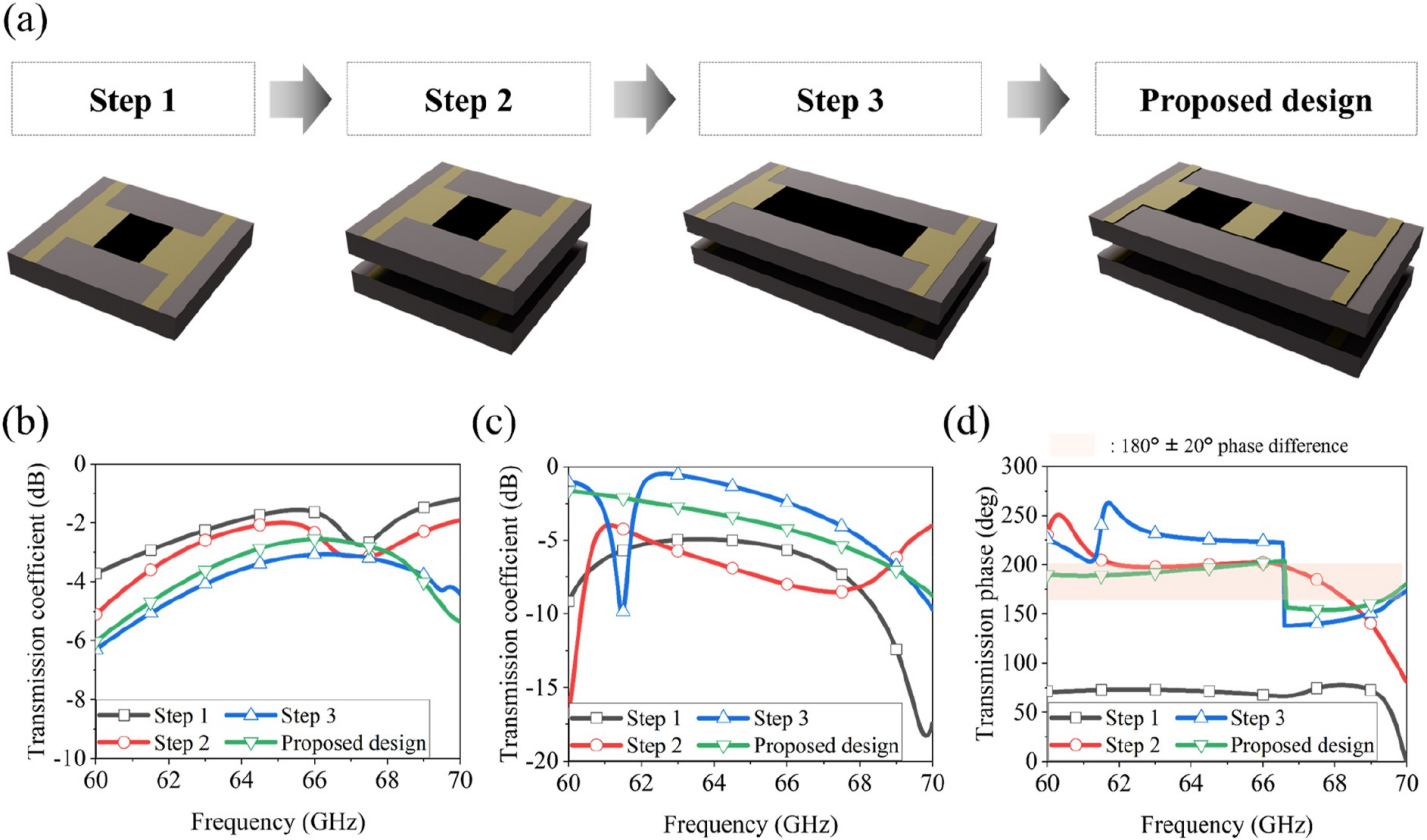
Design of the proposed phase‐transition‐based VO_2_ TRIS unit cell and corresponding simulation results. (a) Sequential design stages of the proposed TRIS unit cell. (b, c) Simulated transmission coefficients of the TRIS unit cell across design stages with the VO_2_ switch in the ON and OFF states. (d) Simulated transmission phase difference between the VO_2_ switch in the ON and OFF states across design stages.

### 2.3 Mechanism of Phase Variation Based on VO_2_ Switch Dimensions

As detailed previously, adjusting the VO_2_ switch size enables phase variation with low design complexity. To investigate this effect, a parametric study was conducted on the transmission coefficient and phase response as functions of the VO_2_ switch length $v_{x}$. Figure 5a illustrates the transmission coefficient for the ON and OFF states as a function of $v_{x}$. At $v_{x}$ = 1 mm, the VO_2_ switch exhibits band‐stop characteristics at the target frequency in both the ON (10 Ω/sq) and OFF (12,000 Ω/sq) states. Increasing $v_{x}$ to 1.5 mm improves the transmission coefficient in both states. However, at $v_{x}$ = 2 mm, it decreases by approximately 1 dB, particularly in the ON state, owing to increased ON‐state resistance, which reduces switching efficiency. Consequently, optimal transmission coefficients in both states are achieved at $v_{x}$ = 1.5 mm. Figure 5b illustrates the transmission phase response as a function of $v_{x}$ in the ON state. As depicted, the phase response becomes stable and ripple‐free at $v_{x}$ = 1.25 mm. As $v_{x}$ increases, the phase‐wrapping region shifts toward lower frequencies, following which it returns toward higher frequencies. This behavior results from inductance changes in the VO_2_ switch induced by variations in $v_{x}$. Figure 5c presents the transmission phase response of the TRIS unit cell with the VO_2_ switch in the OFF state as a function of $v_{x}$. As depicted, the transmission phase variation with $v_{x}$ is negligible. These findings confirm that the desired phase variation can be achieved by adjusting the ON‐state transmission phase through appropriate selection of $v_{x}$. Figure 5d illustrates the transmission coefficient as a function of the VO_2_ switch width $v_{y}$ in both ON and OFF states. At $v_{y}$ = 0.9 mm, a resonance exhibiting band‐stop characteristics emerges in both states. At $v_{y}$ = 1.3 mm, no prominent band‐stop behavior is observed, and the transmission coefficient remains relatively stable. At $v_{y}$ = 1.7 mm, the ON‐state transmission coefficient falls below −15 dB, and multiple band‐stop resonances are also observed in the OFF state. Overall, $v_{y}$ ≤ 1.3 mm yields higher transmission coefficients. Figure 5e presents the transmission phase response of the TRIS unit cell with the VO_2_ switch in the ON state as a function of $v_{y}$. As $v_{y}$ increases, the phase‐wrapping region shifts toward higher frequencies. Whereas variation in $v_{x}$ affects only the phase‐wrapping frequency, changes in $v_{y}$ influence both the wrapping frequency and the transmission phase level. For instance, prior to the phase‐wrapping point, the transmission phase is approximately −175° at $v_{y}$ = 0.9 mm and rises to approximately −30° at $v_{y}$ = 1.7 mm, indicating a gradual increase with increasing $v_{y}$. Figure 5f presents the transmission phase response of the TRIS unit cell with the VO_2_ switch in the OFF state as a function of $v_{y}$. A phase‐wrapping behavior similar to that observed earlier is evident; however, for $v_{y}$ ≥ 1.3 mm, the variation becomes negligible. Based on both the transmission coefficient and phase‐response results, $v_{y}$ = 1.3 mm is identified as the optimal value. Although achieving phase variation is essential for TRIS design, it is typically challenging. The proposed design approach enables this requirement to be met efficiently, thereby reducing the overall design complexity of TRIS structures.

**FIGURE 5:**
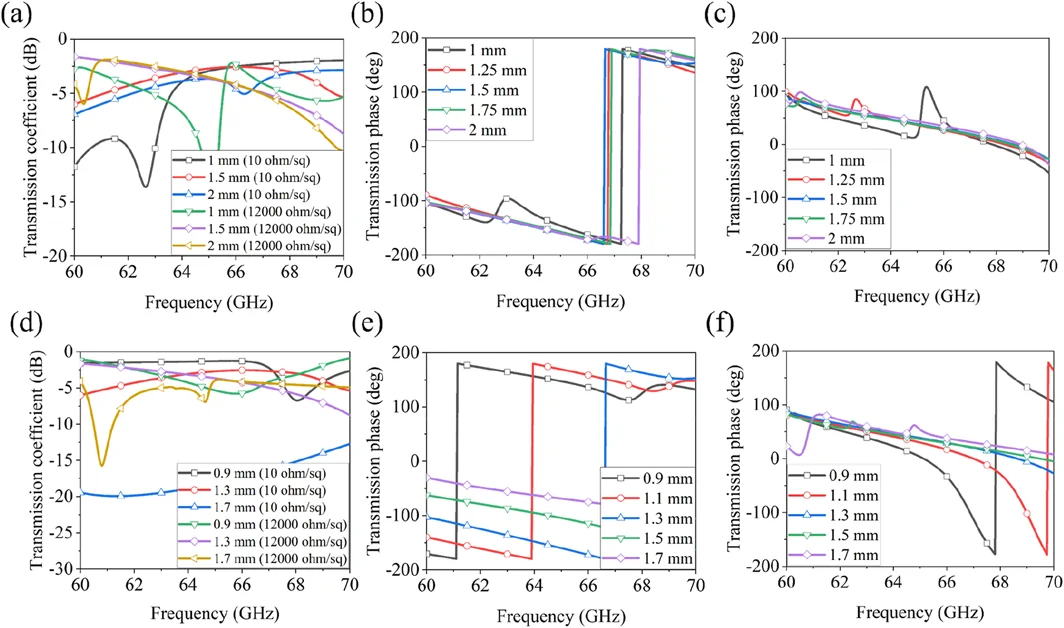
Parameter study of VO_2_ switch geometry variation. (a) Simulated transmission coefficients of the TRIS unit cell with the VO_2_ switch in the ON and OFF states as a function of $v_{x}$. (b, c) Simulated transmission phases of the TRIS unit cell with the VO_2_ switch in the ON and OFF states as a function of $v_{x}$. (d) Simulated transmission coefficients of the TRIS unit cell with the VO_2_ switch in the ON and OFF states as a function of $v_{y}$. (e, f) Simulated transmission phase of the TRIS unit cell with the VO_2_ switch in the ON and OFF states as a function of $v_{y}$.

### 2.4 Thermal‐Crosstalk Analysis

Because the proposed VO_2_‐based TRIS is thermally reconfigured, heat from the activated VO_2_ switches may diffuse through the multilayer structure and increase the temperature of neighboring OFF state switches, potentially causing unintended phase transitions. To evaluate this thermal‐crosstalk effect, transient thermal simulations were performed using ANSYS Icepak. Figure 6a shows the simulation setup. A 2 × 3 array was considered, where the first and third columns were activated and the temperature of the VO_2_ switches in the central OFF state column was monitored. This configuration allows the thermal influence from the neighboring activated columns on the OFF state VO_2_ switches to be evaluated while reducing the computational cost of the thermal simulation. The six outer faces of the surrounding air domain were defined as opening boundaries with an ambient temperature of 20°C. Natural convection, thermal radiation, and the specified flow regime were included in the simulation. The eight VO_2_ switches in each unit cell of the first and third columns were maintained at a fixed temperature of 120°C. Although the fabricated VO_2_ switches exhibit ON state resistance characteristics at approximately 90°C, a higher temperature of 120°C was selected to evaluate thermal crosstalk under a more conservative thermal condition. The thermal conductivities of Rogers RT/duroid 5880 and copper were set to 0.2 and 400 W/m·K, respectively. Figure 6b shows the transient temperature response monitored at the OFF state VO_2_ switch. The temperature rapidly increased from 20°C to approximately 55°C during the first 30 s and then increased gradually. After approximately 70 s, the temperature approached a steady‐state value of about 60°C. Importantly, this temperature remained below the temperature required for the VO_2_ switches to transition to the ON state, even though the activated switches were maintained at the conservative temperature of 120°C. Figure 6c shows the layer‐by‐layer temperature distributions at 80 s. Similar heat‐diffusion patterns were observed across the four layers. The highest temperature in the neighboring OFF state VO_2_ region was approximately 60°C, whereas most of the remaining OFF state region remained at approximately 40°C–50°C. These results indicate that unintended thermal switching of the neighboring OFF state VO_2_ switches is unlikely under the investigated continuous thermal condition.

**FIGURE 6:**
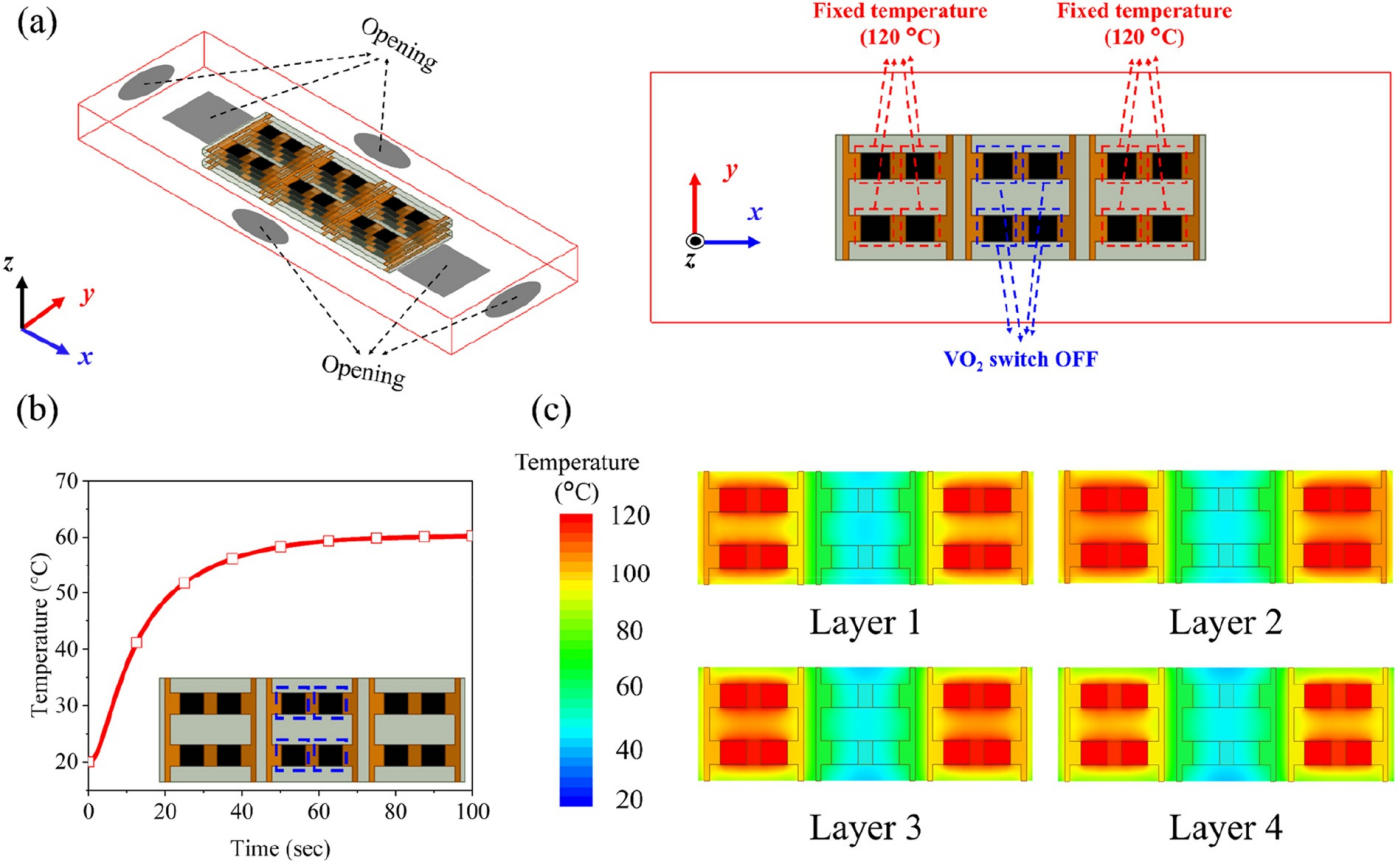
Thermal‐crosstalk analysis of the proposed VO_2_‐based TRIS. (a) ANSYS Icepak simulation setup for a 2 × 3 array, (b) transient temperature response monitored at the adjacent OFF‐state VO_2_ switch, and (c) layer‐by‐layer temperature distributions at 80 s.

### 2.5 Fabrication of the Proposed Phase‐Transition‐Based VO_2_ TRIS

An optimized TRIS comprising a 25 × 21 array of unit cells was fabricated. Column‐wise control was implemented, and bias lines were added to supply DC voltage to the VO_2_ switches (Supporting Information S1: Note 4). Figure 7a illustrates the complete TRIS structure prior to VO_2_ switch printing on the PCB. After forming the copper patterns using a PCB process, VO_2_ ink was screen‐printed into the gaps between the copper patterns. Because the VO_2_ switches had to be printed on both sides of the Rogers 5880 substrate, precise alignment of the switches was essential. To ensure accurate alignment, holes were drilled along the substrate diagonals, and screen‐printed alignment marks were inserted into these holes to match the two sides accurately (Supporting Information S1: Note 5). First, all VO_2_ switches on one side were printed 15 cycles and then dried using a heat gun at approximately 90°C. The substrate was then flipped over and accurately aligned using the alignment marks, after which VO_2_ ink was screen‐printed 15 cycles on the opposite side. After the VO_2_ switches were printed on both sides, the substrate was thermally sintered in an oven at 120°C for 30 min. This procedure was adopted to ensure uniform thermal sintering conditions for the VO_2_ switches on all layers. Figure 7b depicts the screen‐printed VO_2_ switches in each layer. As evident from the images, the VO_2_ switches are accurately positioned across all layers. Figure 7c,d present the side and top views, respectively, of the final VO_2_‐based TRIS prototype. As depicted, the VO_2_‐switch‐printed substrate was integrated with the 3D printed frame, and the final prototype was completed by soldering lead wires to the pads at the ends of the bias lines. The 3D‐printed air‐gap frame was then positioned between the Rogers 5880 substrates. The 3D‐printed air‐gap frame was printed in a square‐loop shape to minimize its influence on the TRIS unit cells. The gap size of the fabricated frame was measured to be 0.56 mm, and it was confirmed that this deviation had no significant effect on the performance of the optimized VO_2_‐based TRIS (Supporting Information S1: Note 6).

**FIGURE 7:**
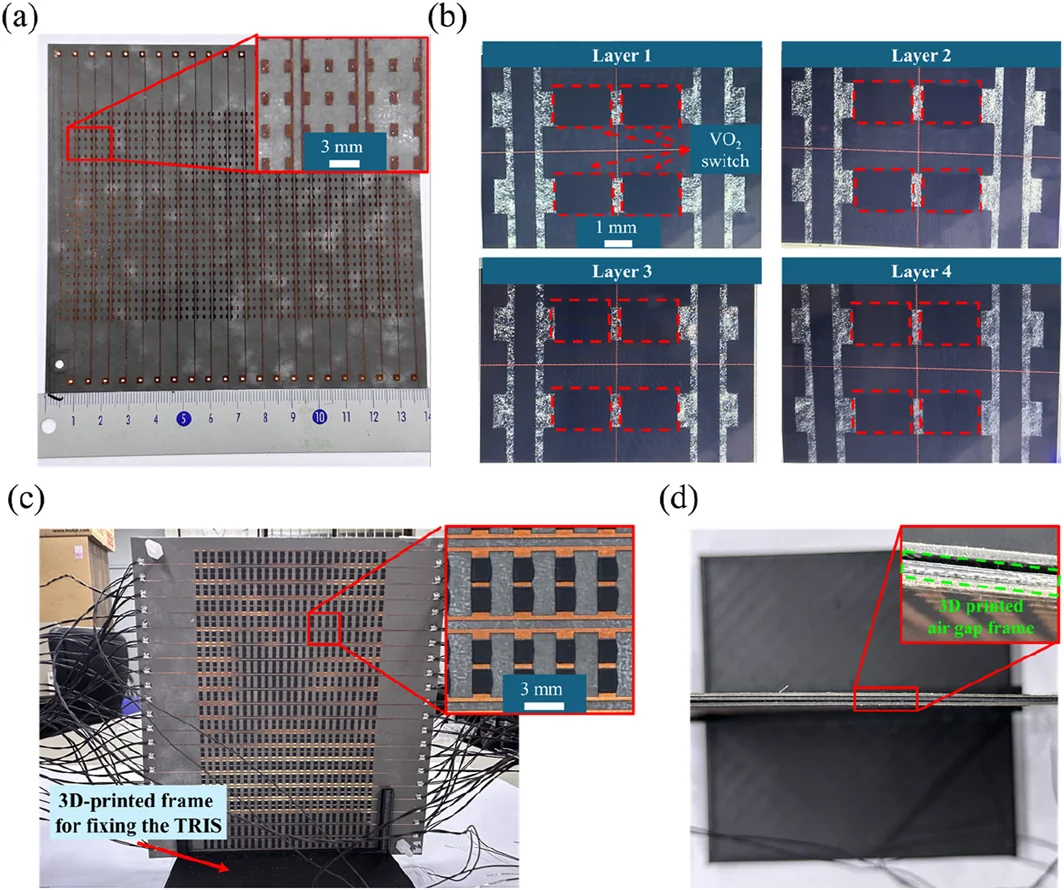
Fabrication process of the proposed phase‐transition‐based VO_2_ TRIS. (a) Full structure without VO_2_ switches fabricated using a PCB process. (b) Microscope image of screen‐printed VO_2_ switches on each layer. (c, d) Side and top views of the fabricated VO_2_ TRIS prototype with a 3D printed frame.

### 2.6 Measurement of the Fabricated Phase‐Transition‐Based VO_2_ TRIS

We evaluated the performance of the fabricated phase‐transition‐based VO_2_ TRIS. Before its fabrication, we performed full‐structure simulations under perfect magnetic conductor boundary conditions (Supporting Information S1: Note 7). The main beam direction of the transmitted wave was determined by controlling the parameters in the array factor (AF) equation. Notably, for a linear TRIS array, the AF is defined as follows:

(1) $$\left|\text{AF}\left(\theta \right)\right|=\sum_{n=1}^{N}\exp\left[j\left(kx_{n}\;\sin\;\theta +\phi_{n}\right)\right],$$

where k is the wave number, $x_{n}$ is the position of the *n*th unit cell, and $\phi_{n}$ is the phase state assigned based on the coding sequence. The spatial position of each unit cell is given by the following equation:

(2) $$x_{n}=\left(n-\frac{N+1}{2}\right)p_{x},$$

where N is the number of columns and $p_{x}$ is the physical unit‐cell pitch along the *x*‐direction. Here, the effective phase‐gradient interval D can be defined as follows:

(3) $$D=Mp_{x},$$

where M is the number of consecutive unit cells assigned to the same coding state.

(4) $$s\text{in}\theta_{t}=\frac{2\gamma +1}{2}\;\frac{\lambda }{D},$$

where γ is the order of the grating lobe in an array antenna. In the fundamental mode, γ in Equation (4) is zero, and the transmission angle is determined by the effective phase‐gradient interval D. At 65 GHz, when M = 1, the effective phase‐gradient interval D is 6.3 mm, and Equation (4) gives a transmission angle of approximately ± 21°. When M = 2, the effective phase‐gradient interval D increases to 12.6 mm, resulting in a transmission angle of approximately ± 10.5°. Since the minimum possible value of M is 1 in the proposed TRIS, the minimum effective phase‐gradient interval D is 6.3 mm, corresponding to a maximum achievable beam transmission angle of approximately ± 21°. Code ‘0’ corresponds to a VO_2_ sheet resistance of 12,000 Ω/sq, whereas code ‘1’ corresponds to a VO_2_ sheet resistance of 10 Ω/sq. Because each coding sequence produces a distinct spatial distribution of $\phi_{n}$, the constructive interference condition, and thus the main lobe direction, is determined by the angle θ that maximizes |AF(θ)|. Our simulation results confirm that the proposed TRIS, using coding combinations from 0000 to 00001111, steers the transmitted beam over a range of 0° to ± 21° (Supporting Information S1: Note 8 and 9).

Figure 8a illustrates the biasing configuration and electrical connections of the fabricated TRIS. As depicted, the +DC terminal was connected to the right side of the structure, whereas the −DC terminal was connected to the left. When voltage was applied, current flowed through the VO_2_ switches, as illustrated in Figure 8b. According to Joule's law, the electrical energy supplied to the VO_2_ switch is converted into thermal energy, thereby triggering the ON state. As the applied voltage increased from 0 V, the temperature of the VO_2_ switch rose. A sharp temperature increase occurred when the voltage rose from 13 to 13.5 V, during which the temperature jumped from 50°C to above 100°C (Supporting Information S1: Note 10). This abrupt rise resulted from a rapid drop in resistance caused by the phase transition of the VO_2_ switch. Figure 8c displays photographs of the TRIS measurement setup. During this measurement, a V‐band horn antenna was used as both the transmitting (Tx) and receiving (Rx) antennas, and a performance network analyzer was used to measure the transmission coefficient in both the ON and OFF states of the TRIS. The VO_2_ switches were controlled using a DC power supply. Figure 8d illustrates a schematic of the measurement setup. As depicted, the Tx antenna was fixed facing the TRIS, whereas the Rx antenna was rotated from 0° to 30°. Meanwhile, both antennas were positioned symmetrically on opposite sides of the TRIS, with each antenna placed 0.4 m away from the TRIS. Although the 0.4 m distance does not satisfy the conventional far‐field criterion, the measurement was used to evaluate the relative transmission enhancement and principal beam‐steering behavior of the fabricated TRIS under the available bistatic configuration. Similar finite‐distance bistatic measurements have been employed to experimentally validate RIS and metasurface prototypes [31]. Accordingly, the measured results should be interpreted as verification of the relative angular response and beam‐steering functionality rather than as an ideal plane‐wave far‐field characterization. The angular measurement range of the Rx antenna was limited to 30° by the mechanical constraints of the measurement setup. The amplitude and phase characteristics of the fabricated TRIS were measured. Figure 8e shows the measured transmission coefficients in the ON and OFF states. To maintain the VO_2_ switches in the ON state during the measurement, the fabricated TRIS was heated using a hot‐air gun. In the ON state, the transmission coefficient increased from approximately −8 dB to −3 dB as the frequency increased. The measured response was shifted by approximately 1 GHz toward a higher frequency and exhibited approximately 2–4 dB higher loss than the simulated result, while maintaining a similar overall frequency response. In the OFF state, the transmission coefficient decreased from approximately −2.5 dB to −7 dB as the frequency increased. Although an additional loss of approximately 1 dB was observed compared with the simulation, the measured response showed a similar trend. The discrepancies between the simulated and measured results may be attributed to fabrication tolerances, resistance variations caused by the nonuniform thickness of the screen‐printed VO_2_ patterns, and assembly errors introduced during the integration of the fabricated TRIS layers. Figure 8f shows the measured transmission phases in the ON and OFF states. In the OFF state, the transmission phase decreased from approximately 228°–188° over the measured frequency range, whereas in the ON state, it decreased from approximately 32°–9.8°. The resulting phase difference between the two states ranged from approximately 174°–196° over 60–67 GHz. Figure 8g–i illustrate the measured transmission coefficients of the TRIS for selected coding sequences in the ON and OFF states. For code 0101, which steers the transmitted beam to ± 20° according to the AF equation, the Rx antenna was fixed at 20°. Across the 64–66 GHz band, the ON state of the TRIS demonstrated an improvement of approximately 15 dB over the OFF state. For code 0011, corresponding to a steering angle of ± 11°, the Rx antenna was positioned at 10°, and a similar improvement of around 15 dB was observed. For code 00001111, which produces a steering angle of ± 5°, the Rx antenna was set at 5°. In this case, the transmission enhancement was approximately 5 dB because the reference level remained relatively high near the boresight direction. The transmission enhancement at small steering angles is lower than that at larger steering angles because the improvement is evaluated relative to the code 0000 state, whose response is strongly influenced by the angular radiation pattern of the feed horn antenna. In the horn radiation pattern, the gain at 5° remains relatively close to that at 0°, whereas the gain decreases substantially at 20°. Therefore, the reference level of code 0000 is relatively high at small observation angles, resulting in a smaller difference between the beam‐steered state and the reference state. Consequently, the transmission enhancement appears smaller at small steering angles than at larger angles. Thus, the apparently smaller improvement at small steering angles primarily reflects the angular dependence of the reference state rather than a substantial reduction in the beam‐steered transmission level.

**FIGURE 8:**
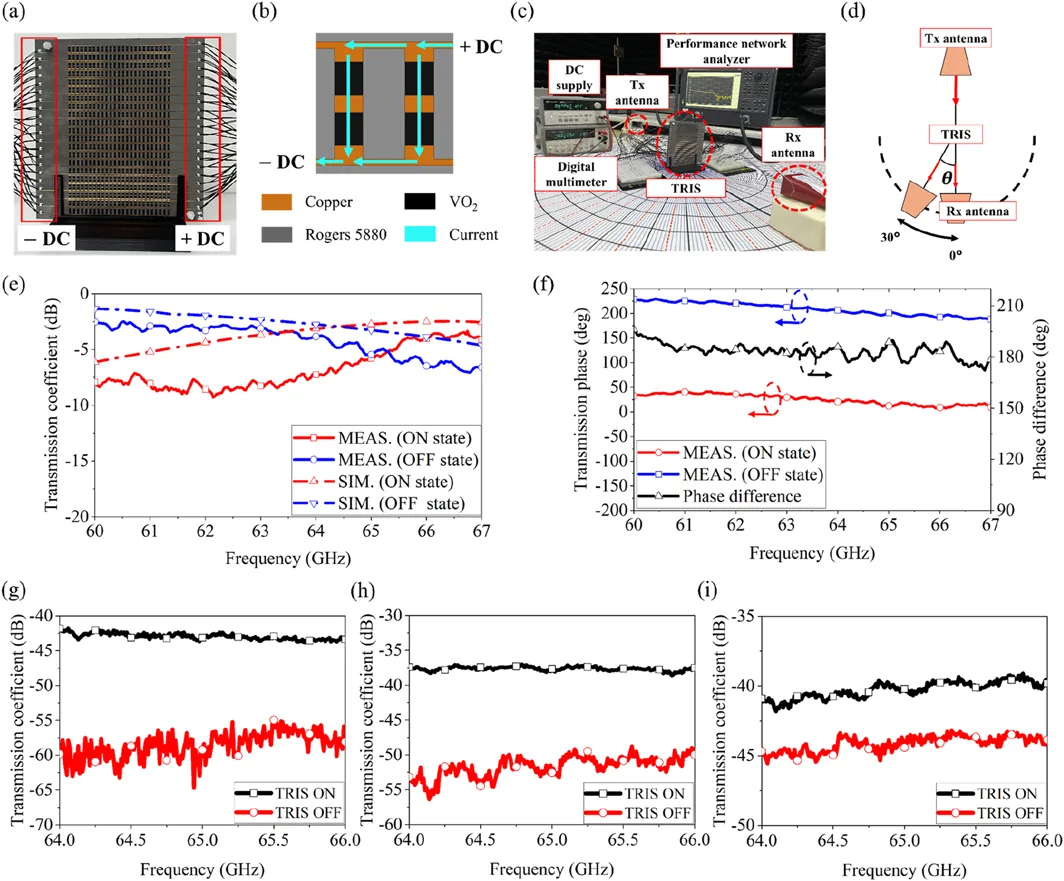
Measurement setup and results. (a) DC biasing configuration of the VO_2_‐based TRIS, illustrating the column‐wise negative (−DC) and positive (+DC) bias connections. (b) Current flow in the ON state of the VO_2_ switch. (c) Photographs of the measurement setup. (d) Schematic of the measurement setup. (e) Measured and simulated transmission coefficients of the fabricated TRIS in the ON and OFF states and (f) measured transmission phases and the corresponding ON–OFF phase difference. (g–i) Measured transmission coefficients for codes 0101, 0011 and 00001111 when the TRIS is in the ON and OFF states.

Finally, the transmission coefficient for each code was measured across angles from 0° to 30° and compared with full‐wave simulations. Figure 9a–d compare the normalized scattering patterns obtained from simulations and measurements for each coding configuration at 65 GHz. The angles were measured at 5° intervals between 0° and 30°, and angles exceeding 30° could not be measured due to limitations of the measurement device. As shown in Figure 9a, code 0000 exhibits maximum radiation at 0° at 65 GHz, which closely matches the simulation results. Figure 9b compares the results for code 0101. At 65 GHz, the maximum beam direction was 21° in the simulation and 20° in the measurement. The overall trend was similar to the simulation, with the 1° discrepancy arising from the 5° measurement resolution. Figure 9c corresponds to code 0011. Similar to the previous case, the maximum beam direction at 65 GHz shows a slight difference between the simulated value of 10.5° and the measured value of 10°. The measured level between 0° and 5° exceeds the simulated result, likely due to a stronger intermediate beam component. Figure 9d corresponds to code 00001111. At 65 GHz, the maximum beam direction shows a difference of approximately 1° between simulation (4°) and measurement (5°), but the overall trend remains consistent. Some measured scattering magnitudes in Figure 9 are higher than the corresponding simulated values. A similar tendency was observed in our previous sub‐terahertz IRS study, where additional bias and supporting structures were found to modify the scattering response and increase the magnitude at certain observation angles [35]. In the present study, the simulated results in Figure 9 were obtained without fully considering the additional bias lines used in the fabricated TRIS. Therefore, parasitic scattering from these practical bias structures may partly account for the locally higher measured magnitudes. In addition, because absorbers were not placed around the TRIS during the measurement, the influence of diffraction from the feed antenna cannot be completely excluded. These effects may together contribute to the remaining discrepancy between the simulated and measured results. Overall, the measured results agree well with the simulations, supporting the proposed TRIS as a low‐cost hardware platform with potential for mm‐wave beam‐control applications.

**FIGURE 9:**
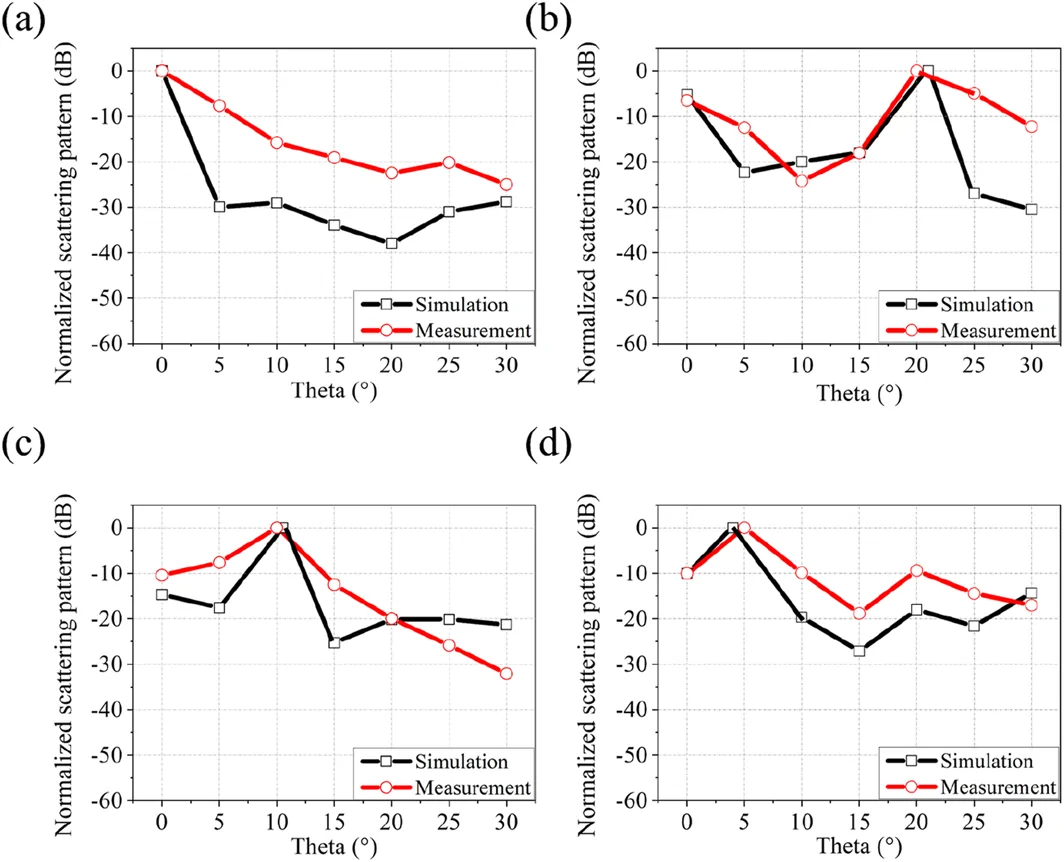
Comparison of the normalized simulated and measured scattering patterns at 65 GHz for (a) code 0000, (b) code 0101, (c) code 0011 and (d) code 00001111.

The experimental Tx/Rx distance of 0.4 m does not satisfy the conventional Fraunhofer far‐field criterion for the fabricated TRIS. To examine the sensitivity of the observed beam‐steering response to the measurement distance, additional full‐structure simulations were performed using the model shown in Figure 10a. Two configurations with Tx/Rx distances of 0.4 and 1 m were considered, as illustrated in Figure 10b, using the same horn‐antenna characteristics as those employed in the experiment. Figure 10c compares the simulated angular transmission responses for code 0101. In both configurations, the maximum transmission level occurs at approximately 20°, and the overall angular responses exhibit similar trends. These results indicate that the principal beam‐steering direction observed at the experimental distance is maintained when the observation distance is increased. Nevertheless, the present measurements do not constitute a standard far‐field characterization, and therefore absolute far‐field gain, sidelobe level, and half‐power beamwidth are not quantitatively evaluated in this work. To evaluate the thermal‐cycling stability of the screen‐printed VO_2_ switching structure, a representative single‐layer test sample was fabricated using the same substrate, VO_2_ pattern geometry, film thickness, and fabrication process as those of the proposed TRIS. The sample was repeatedly cycled between room temperature and 100°C using a hot plate, and the resistance of one column was measured in the ON and OFF states to monitor possible electrical degradation caused by repeated thermal expansion and contraction. Figure 10d shows the normalized column resistance measured at selected cycle counts up to 1000 thermal cycles. The resistance values were normalized to the initial ON‐ and OFF‐state resistances of 2.3 Ω and 1.52 kΩ, respectively. The normalized resistance remained within approximately ± 4% of the initial values at all measured cycle counts, indicating that no significant electrical degradation of the screen‐printed VO_2_ switching structure was observed up to 1000 thermal cycles under the investigated condition. Figure 10e shows infrared (IR) camera images used to measure the thermal crosstalk of the OFF state column located between the ON state columns under code 0101. After heating the ON state columns for 1 min, the temperature at several locations of the OFF state column was monitored for an additional 2 min while the ON state columns remained continuously activated. The measured temperature of the OFF state column ranged from approximately 36°C–45°C. These temperatures are well below the phase‐transition temperature of approximately 67°C for the VO_2_ switches used in this study. Therefore, the OFF state column remained below the temperature required for the VO_2_ phase transition, indicating that thermal crosstalk did not induce an unintended phase transition in the OFF state column.

**FIGURE 10:**
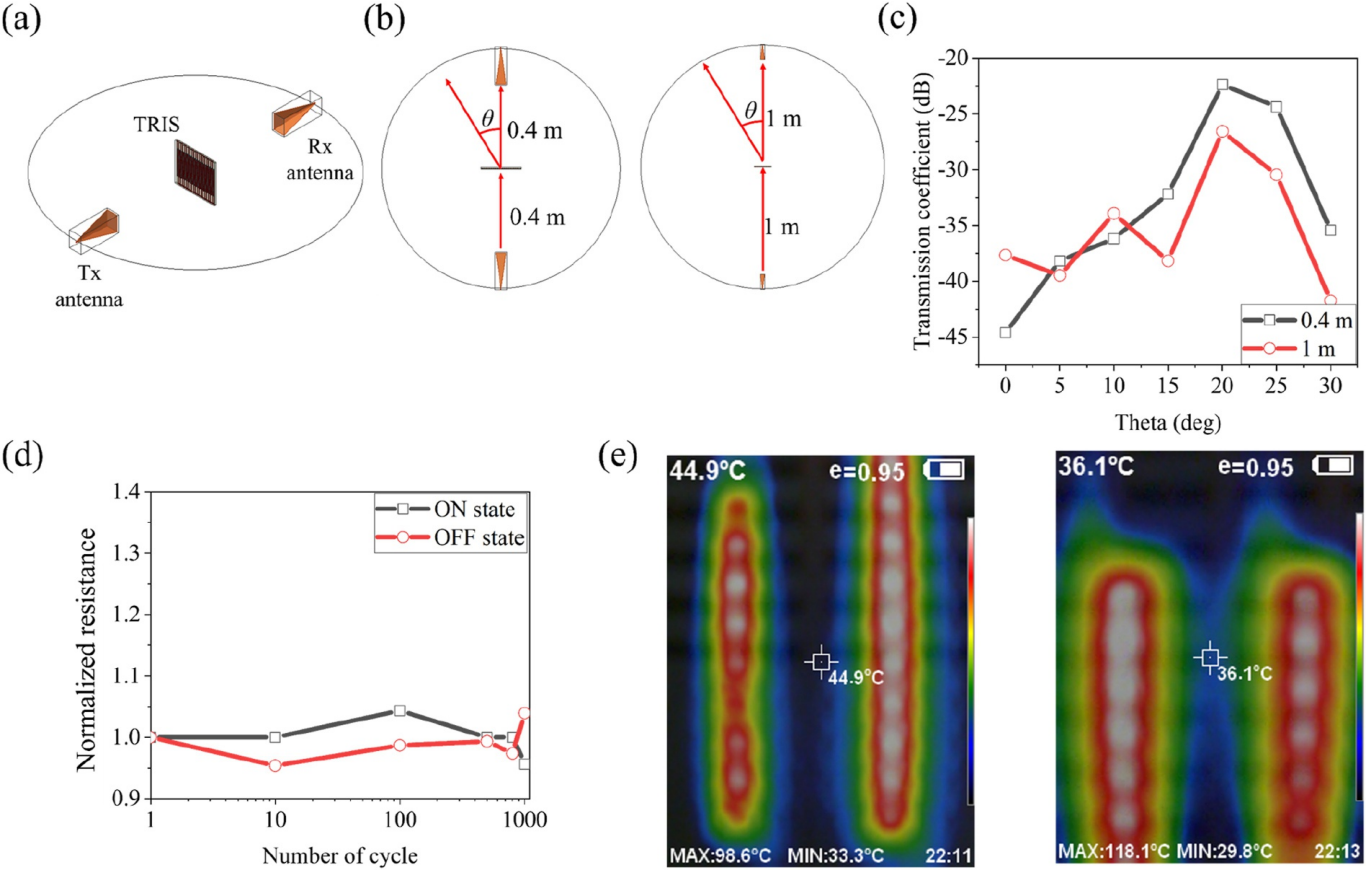
Effects of measurement distance, thermal‐cycling reliability, and thermal‐crosstalk of the proposed TRIS. (a) Full‐structure simulation model reproducing the measurement configuration, (b) simulation configurations with Tx/Rx distances of 0.4 and 1 m, (c) simulated angular transmission responses for code 0101 at the two Tx/Rx distances, (d) normalized column resistance in the ON and OFF states during 1000 thermal cycles, and (e) thermal‐crosstalk measurement results.

## 3 Discussion

Table 1 compares the proposed phase‐transition‐based VO_2_ TRIS with previously reported designs. For instance, Pan et al. [27] proposed a PIN‐diode‐based TRIS operating at 97 GHz. However, this TRIS demonstrated an element loss of 11 dB, considerable assembly complexity owing to soldering and high fabrication cost. These findings underscore the limitations of employing PIN diodes in the mm‐wave band. Further, Shen et al. [23] developed a TRIS operating at 100 GHz using Schottky diodes. Although this design achieved considerably improved performance relative to earlier designs, including a low insertion loss of 2.8 dB and a scanning angle of ± 60°, its reliance on Schottky diodes resulted in high fabrication cost, and the use of conductive adhesive increased fabrication complexity. Another research team proposed a TRIS fabricated based on complementary metal‒oxide‒semiconductor (CMOS) technology and liquid crystals [36, 37]. Although this CMOS technique offered benefits such as fast switching speed, low power consumption, and precise phase control, the associated fabrication was both costly and complex. In parallel, although liquid–crystal‐based TRISs could be realized at relatively low material costs, their fabrication and assembly processes remain complex. In contrast, the VO_2_‐based TRIS presented here exhibits an element loss of 5.2 dB. The VO_2_ switch reduces design complexity, whereas screen printing further simplifies fabrication. In addition, the fabricated sample was realized with a large aperture size of 28*λ*_0_ × 17.4*λ*_0_ which is larger than those reported in previous studies. In general, a larger RIS aperture is required because the efficiency of an RIS increases with its physical size [38]. However, conventional approaches mainly rely on soldering or conductive adhesives, which can increase parasitic losses or fabrication errors when scaling up the aperture size. In contrast, the screen‐printed VO_2_ switches can be implemented without solder or conductive epoxy, allowing the TRIS to be fabricated with a more idealized structure. Although the aperture size in this work was limited by the available printing equipment, the same fabrication approach can potentially be extended to larger apertures by using a screen printer with a larger printable area. In addition to its scalability, the proposed screen‐printed approach provides a low tuning‐element cost compared with discrete semiconductor‐based implementations. Approximately 3 g of VO_2_ ink is obtained from one preparation, which is sufficient to print one patterned layer of the fabricated TRIS. Four preparations are therefore required for the four‐layer structure, corresponding to a total VO_2_ material cost of approximately $26. For the 525‐unit‐cell prototype, this corresponds to an estimated VO_2_ tuning‐material cost of approximately $0.05 per unit cell. Although eight VO_2_ switching regions are employed in each unit cell, they are simultaneously patterned by screen printing rather than individually purchased and assembled as discrete components. Therefore, the tuning‐element cost remains substantially lower than that of the diode‐based approaches compared in Table 1. The values in Table 1 represent only the estimated tunable‐material/device cost and exclude substrate, fabrication, assembly, and peripheral‐control costs.

**TABLE 1:** Comparison of the proposed phase‐transition‐based VO_2_ TRIS with previous studies on TRISs.

Ref.	Freq. (GHz)	Element loss (dB)	Tuning method	Aperture size (*λ*_0_ × *λ*_0_)	Number of unit cells	Integration method	Scanning range	Estimated tuning‐material/device cost per unit cell	Power consumption (mW/unit cell)^e^
[27]	97	11	PIN‐diode	16.5 × 1	32 × 2	Soldering	—	$13.3^a^	13.6^f^
[23]	100	2.8	Schottky diode	4.3 × 3.2	8 × 8	Conductive adhesive	± 60°	$9.3^b^	N/A
[36]	300	5	Complementary metal‒oxide‒semiconductor	3.4 × 3.4	24 × 24	N/A	± 30°	N/A	0.00168
[37]	408	6	Liquid crystal	21 × 21	48 × 48	N/A	± 30°	<$0.01^c^	N/A
This work	65	5.2	**VO**_**2**_ **switch**	**28** **×** **17.4**	25 × 21	**Screen‐printing**	± 20°	**$0.05** ^d^	151

*Note:* The bold values were used to highlight the key results of our research.

^a^ The unit cost of the MA4AGFCP910 PIN diode was estimated based on the 500‐piece pricing listed by Mouser Electronics.

^b^ The Schottky‐diode cost was estimated using the unit price of the commercially available MACOM MA4E1317 GaAs Schottky diode as a reference.

^c^ The liquid‐crystal (LC) cost was estimated from the required LC volume and the typical raw‐material cost of high‐birefringence nematic LC mixtures; the exact LC composition and commercial price were not reported in Ref. [37].

^d^ The estimated VO_2_ cost includes only the VO_2_ ink material cost and excludes fabrication and assembly costs.

^e^ The power consumption was calculated based on the power required to maintain the ON state per unit cell.

^f^ The power consumption of the PIN diode was estimated from the electrical characteristics provided in the manufacturer's datasheet.

However, several limitations remain. First, the beam‐scanning range of the proposed TRIS is constrained by the relatively large electrical size of the unit cell. In 1‐bit space‐coded RISs, the achievable steering angle is strongly dependent on the spatial period of the coding structure [23, 35]. In the present design, the VO_2_ switch dimensions were increased to simultaneously achieve a sufficiently high transmission magnitude and an approximately 180° transmission‐phase difference, which inevitably enlarged the unit‐cell period and limited the achievable steering range. At 65 GHz, the unit‐cell period along the beam‐steering direction is 6.3 mm, corresponding to approximately 1.36*λ*_0_. According to the AF relation, this electrical period results in a maximum steering angle of approximately ± 21°, which is consistent with the full‐wave simulation results. Therefore, substantially wider beam steering cannot be achieved using the present unit‐cell dimensions and coding architecture. To extend the scanning range, future designs should reduce the electrical size of the unit cell while maintaining an approximately 180° transmission‐phase difference and sufficiently low transmission loss. Potential approaches include phase‐shifter‐integrated structures [15], Huygens‐response‐based unit‐cell topologies [22], and Pancharatnam–Berry‐phase‐based structures [23], which could provide the required phase control with a more compact electrical size and thereby enable a wider beam‐scanning range.

Second, the proposed VO_2_‐based TRIS exhibits relatively high transmission loss. This loss can be reduced by improving both the electrical properties and the geometry of the printed VO_2_ switches. As shown in Figure 2d, the practical VO_2_ switch introduces additional losses of approximately 3 and 1 dB at 65 GHz in the ON and OFF states, respectively, compared with the ideal case. Therefore, using printed VO_2_ with a lower ON‐state resistance through alternative or optimized fabrication processes could improve the transmission performance [28, 39]. In addition, the additional loss observed in the OFF state indicates that a higher OFF‐state resistance is also desirable. Accordingly, increasing the ON/OFF resistance ratio of the VO_2_ switch by simultaneously reducing the ON‐state resistance and increasing the OFF‐state resistance could further reduce the transmission loss [26, 40]. The switch resistance can also be optimized by adjusting its dimensions according to the following equation:

(5) $$R=R_{s}\frac{l}{w},$$

where $R_{s}$ is the sheet resistance of the VO_2_ film, l is the current‐path length, and w is the width of the VO_2_ switch. Thus, reducing l or increasing w provides another approach to reducing transmission loss. Conversely, if the transmission loss in the OFF state becomes greater than that in the ON state because of insufficient OFF state resistance, the OFF state resistance can be increased by reducing w and increasing l. Therefore, the geometric optimization of the VO_2_ switch can be used not only to reduce the ON state resistance but also to achieve a better trade‐off between the ON and OFF state transmission characteristics, although the unit‐cell phase response should be reoptimized accordingly. A further limitation stems from the use of a four‐layer FSS‐based unit cell structure. This configuration was adopted to ensure stable thermal isolation of the VO_2_ switches. However, it increases the number of switches and compromises the inherent low‐power advantage of RISs. The fabricated TRIS required 13.5 V and 0.28 A per activated column to induce the VO_2_ phase transition, corresponding to approximately 3.78 W per activated column. The maximum power consumption of the TRIS occurs for code 1111. Approximately 79.4 W is required to maintain all 21 columns in the ON state. For the representative 0101 coding state, 10 of the 21 columns are activated, resulting in a total driving power of 37.8 W. When normalized by the total 525 unit cells, this corresponds to an aperture‐averaged power consumption of approximately 72 mW per unit cell for this coding state. The relatively high static power consumption is an important limitation of the present TRIS. It originates from the thermally driven VO_2_ switching mechanism and is further increased by the four‐layer architecture, which requires eight VO_2_ switching regions per unit cell. Future work should therefore focus on reducing the number of layers and switching elements and improving the electrical and thermal efficiency of the switching architecture. For example, a simplified VO_2_‐based unit‐cell architecture inspired by the dual‐layer configuration reported in Ref. [22] could reduce both the required driving power and the complexity of the bias network. Combined with a two‐dimensional matrix‐addressable bias architecture, it could enable independent control of individual unit cells or two‐dimensional subarrays, allowing the required phase distributions to be formed along both the *x*‐ and *y*‐directions. For thermally driven VO_2_ switches, localized thermal control and sufficient thermal isolation between neighboring cells would also be required to minimize thermal crosstalk. This approach could provide a practical route toward two‐dimensional beam scanning.

The present work focuses on the electromagnetic characteristics and experimental beam‐steering performance of the proposed TRIS at 65 GHz. A complete end‐to‐end communication‐system evaluation is beyond the scope of the present study. In practical mm‐wave links, communication performance can additionally be affected by phase‐quantization errors, fabrication‐induced phase and amplitude errors, and multipath propagation. Therefore, future work should evaluate the proposed TRIS using modulated waveforms and system‐level metrics such as error vector magnitude (EVM), bit error rate (BER), and achievable rate or channel capacity. Comparisons with and without the TRIS under representative indoor propagation conditions would provide a quantitative assessment of its communication‐level benefit.

Another important direction for future work is the integration of adaptive closed‐loop control with the proposed TRIS. The present study focuses on the hardware implementation and electromagnetic beam‐steering capability of the TRIS, and predefined coding states were used for experimental validation. However, in practical mm‐wave communication environments, channel conditions and user locations can vary dynamically. Previous studies have demonstrated adaptive beam‐control approaches based on RIS‐aided channel estimation and localization, deep reinforcement learning, robust neural‐network‐based beamforming, and scenario‐adaptive meta‐learning [41, 42, 43, 44]. In a future closed‐loop implementation, channel or user‐location information could be acquired by the communication system and supplied to an external controller, which could adaptively select an appropriate coding state of the TRIS. Such integration would extend the proposed hardware platform toward adaptive beam control in dynamically varying mm‐wave environments.

## 4 Conclusions

In summary, this study presents a low‐complexity phase‐transition‐based TRIS employing a VO_2_ switch. Although previous TRIS designs relied on semiconductor devices such as diodes, they substantially increased fabrication costs and introduced considerable design challenges. In particular, given the fixed physical dimensions of diodes, both the transmission coefficient and phase shift had to be controlled exclusively through modifications to the conductive pattern. In contrast, using a VO_2_ switch allows phase tuning through adjustments to both the conductive pattern and the switch's physical dimensions, thereby reducing design complexity. The proposed TRIS is fabricated using standard PCB processes, screen printing and 3D printing, which significantly lowers fabrication complexity compared with techniques requiring soldering or conductive adhesives. Notably, because high‐efficiency RIS structures typically require large‐area fabrication, such methods tend to complicate fabrication complexity. In this study, we used a screen printer with a limited area of 140 × 140 mm^2^ to fabricate a 25 × 21 TRIS prototype. Notably, employing a screen printer capable of handling larger areas allows more efficient fabrication of large‐area TRIS structures than alternative methods. This approach therefore offers strong potential for low‐cost large‐area TRIS implementations in the mm‐wave band.

## 5 Experimental Section/Methods

*VO*_*2*_ *microparticle* (*μP*) *and ink synthesis*: VO_2_ µPs were synthesized through a hydrothermal process. First, oxalic acid (12.6 g, > 99%, Sigma‒Aldrich) was dissolved in 630 mL of deionized (DI) water, which was followed by the addition of V_2_O_5_ powder (2.1 g, > 98%, Sigma‒Aldrich). The mixture was stirred thoroughly to form a homogeneous yellow slurry, which was then transferred to two 500 mL polypropiolactone‐lined hydrothermal autoclave reactors and heated at 260°C for 6 h. After the reaction, the autoclaves were allowed to cool to room temperature. The resulting blue‐black precipitate was collected via centrifugation at 5000 rpm for 3 min, washed repeatedly with DI water and ethanol and dried in a vacuum oven (SH‐VDO‐PK‐S1, SH SCIENTIFIC) at 70°C for 1.5 h. The dried powder was subsequently annealed at 390°C for 3 h under vacuum environment to yield the final VO_2_ µPs. To prepare the ink formulation, ethyl cellulose (Sigma‒Aldrich), terpineol (Sigma‒Aldrich), and ethanol were mixed in a 1:4:0.4 weight ratio. The screen‐printable VO_2_ ink was prepared by blending the synthesized VO_2_ µPs with the ink formulation at a 1:1 weight ratio.

*Fabrication and measurement*: Screen printing was performed using an automatic screen printer (SJ‐7320A, SUNGJIN Technology) and a 150 mm × 150 mm stainless steel mesh screen mask (325 mesh count, 22.5° mesh angle, and 8 μm emulsion thickness) for VO_2_ printing. A Sindoh 3DWOX 7X 3D printer was employed to fabricate the supporting frame of the TRIS structure. VO_2_ ink was screen‐printed in 15 cycles on both sides of the TRIS substrate, which had been initially fabricated without VO_2_ switches via a PCB process. The printed layers were then dried in an oven at 120°C for 30 min. Following printing, jumper wires were soldered onto the patterned pads to supply the required DC bias. The 3D‐printed frame was subsequently assembled with the printed substrate to complete the TRIS prototype. Measurements on the VO_2_ TRIS were performed using a performance network analyzer (Keysight N5227B, Keysight) with a frequency range of up to 67 GHz. V‐band standard‐gain horn antennas (SGH‐26‐WR15, Anteral) were employed as the transmitting and receiving antennas during these measurements. The heat required to induce the phase transition in the VO_2_ switches was supplied by a DC power supply (Keysight N6705C, Keysight).

## Author Contributions

**Junghyeon Kim:** conceptualization, methodology, visualization, writing – original draft, writing – review and editing, formal analysis, investigation, validation. **Sumin Bark:** conceptualization, data curation, investigation, validation, visualization, writing – original draft, methodology, formal analysis. **Minjae Lee:** software, data curation, formal analysis, visualization, methodology, validation. **Sungjoon Lim:** conceptualization, funding acquisition, supervision, project administration, writing – review and editing.

## Funding

This research was supported by the National Research Foundation of Korea (NRF) grant funded by the Korea government (MSIT) (RS‐2024‐00405510, RS‐2025‐23323906, RS‐2026‐25472008).

## Conflicts of Interest

The authors declare no conflicts of interest.

## Supporting information


Supporting Information S1

## Data Availability

The data that support the findings of this study are available from the corresponding author upon reasonable request.
