# Supplementary material for: Low‐Complexity Phase–Transition‐Based VO2 Transmissive Reconfigurable Intelligent Surface for Indoor Millimeter‐Wave Communications
Source: Nanophotonics. 2026 Sep 8;15(17):e70291. doi: 10.1002/nap2.70291 (PMC13672137; doi:10.1002/nap2.70291)
Supplement: Supplementary file 1 [file NAP2-15-e70291-s001.pdf]

Supporting Information

A Low-Complexity Phase-Transition-Based VO_2 Transmissive Reconfigurable Intelligent Surface for Indoor Millimeter-Wave Communications

Junghyeon Kim[†]*, Sumin Bark*[†]*, Minjae Lee and Sungjoon Lim**

[†] These authors contributed equally to this work.

The Supplementary Information includes:

Supplementary Note 1. Boundary Conditions for Unit Cell Simulation
Supplementary Note 2. One- and Two-Switch Unit-Cell Comparison
Supplementary Note 3. Variation of the unit-cell Smith chart with incident angle
Supplementary Note 4. Configuration of DC Bias Pads and Lines
Supplementary Note 5. Alignment Using Screen-Printed Markers
Supplementary Note 6. Effect of Air-Gap Height on TRIS Performance
Supplementary Note 7. Full-Structure Simulation Setup
Supplementary Note 8. Comparison Between Analytical Calculation and EM Simulation
Supplementary Note 9. Comparison Between Co- and Cross-polarization
Supplementary Note 10. Temperature Response of the 21-Column VO_2 Switch Array

Supplementary Note 1. Boundary Conditions for Unit Cell Simulation

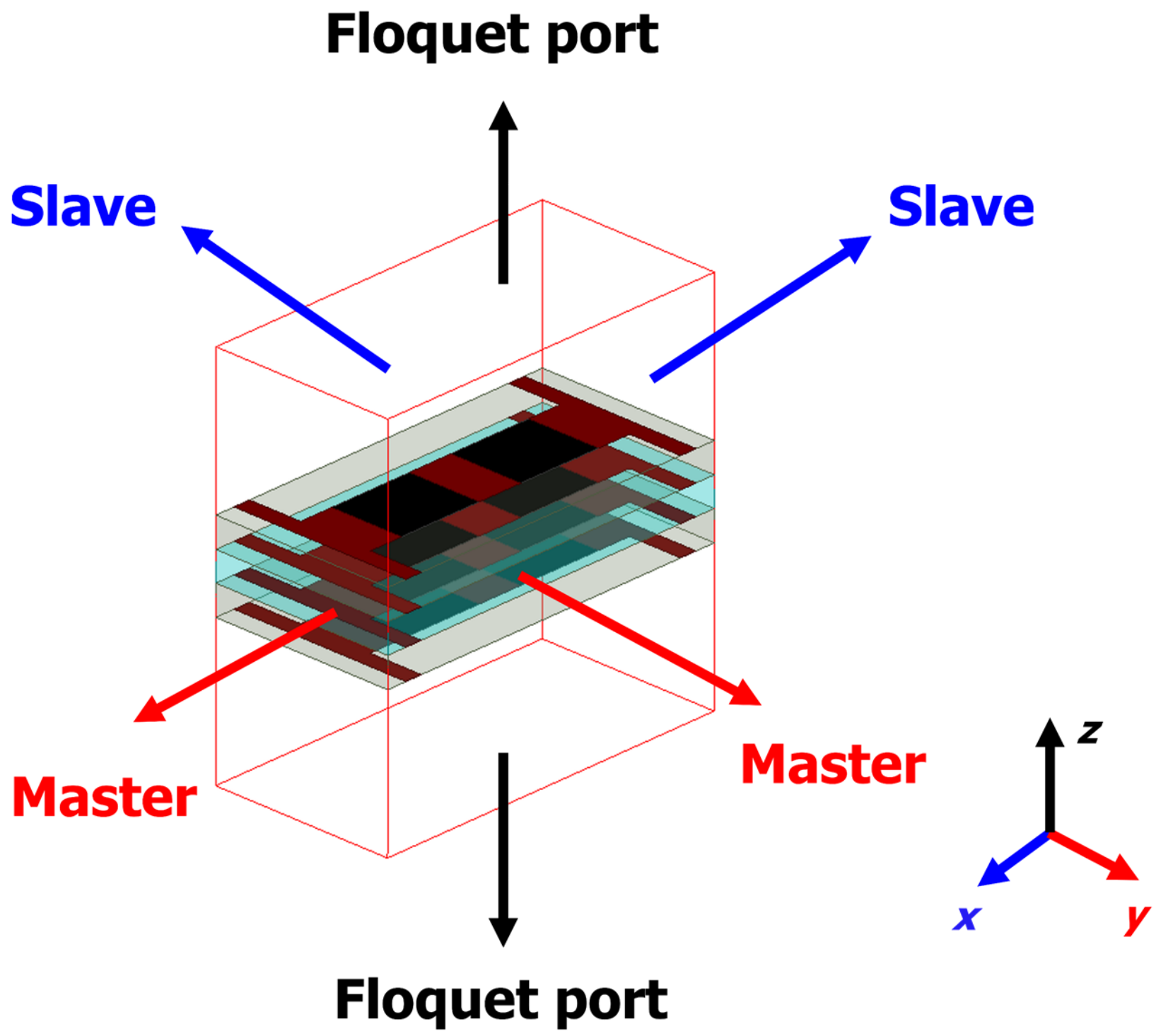

Fig. S1. Unit cell structure simulation setup.

The unit cell simulations for the proposed TRIS were performed using Ansys HFSS (2016). To emulate an infinitely periodic structure, Master/Slave boundary conditions and Floquet ports were applied. The corresponding simulation setup is shown in Fig. S1.

Supplementary Note 2. One- and Two-Switch Unit-Cell Comparison

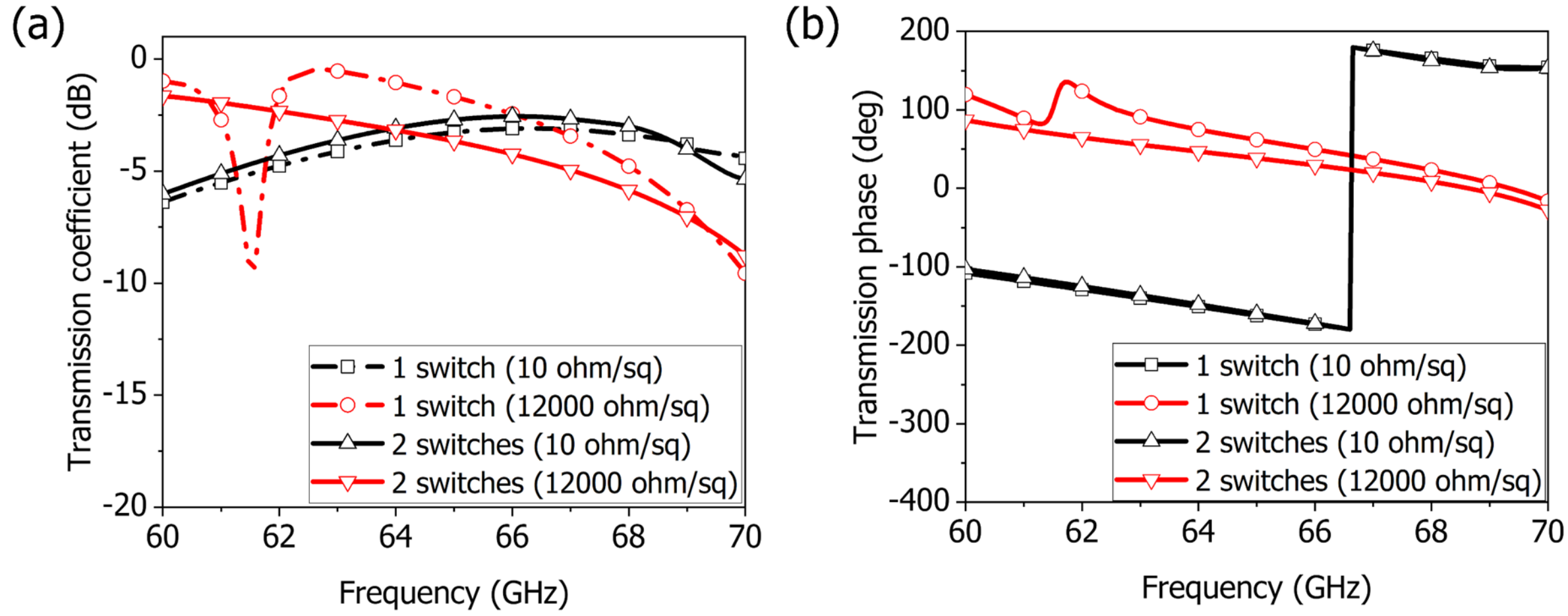

Fig. S2. Comparison of the simulated ON/OFF responses for one-switch and two-switch configurations using VO_2 switches: (a) transmission coefficients and (b) transmission phase results.

For performance comparison, simulations were performed for unit cells containing one VO_2 switch per layer and two VO_2 switches per layer. Fig. S2(a) presents the transmission coefficient results for both configurations in the ON (10 Ω/sq) and OFF (12,000 Ω/sq) states. The results indicate that dividing the switching element into two parts reduces the transmission coefficient in the OFF state while increasing it in the ON state. Since high transmission is required in both states for TRIS operation, the two-switch configuration provides improved performance. Fig. S2(b) shows the corresponding transmission phase responses. The ON-state phase response is nearly identical for both cases, whereas the OFF-state response exhibits a considerable difference. With a single switch, the ON–OFF phase difference exceeds 200°, whereas with two switches it is reduced to approximately 180°. Because a phase difference near 180° is desirable for 1-bit TRIS operation, the two-switch configuration is more suitable.

Supplementary Note 3. Variation of the unit-cell Smith chart with incident angle

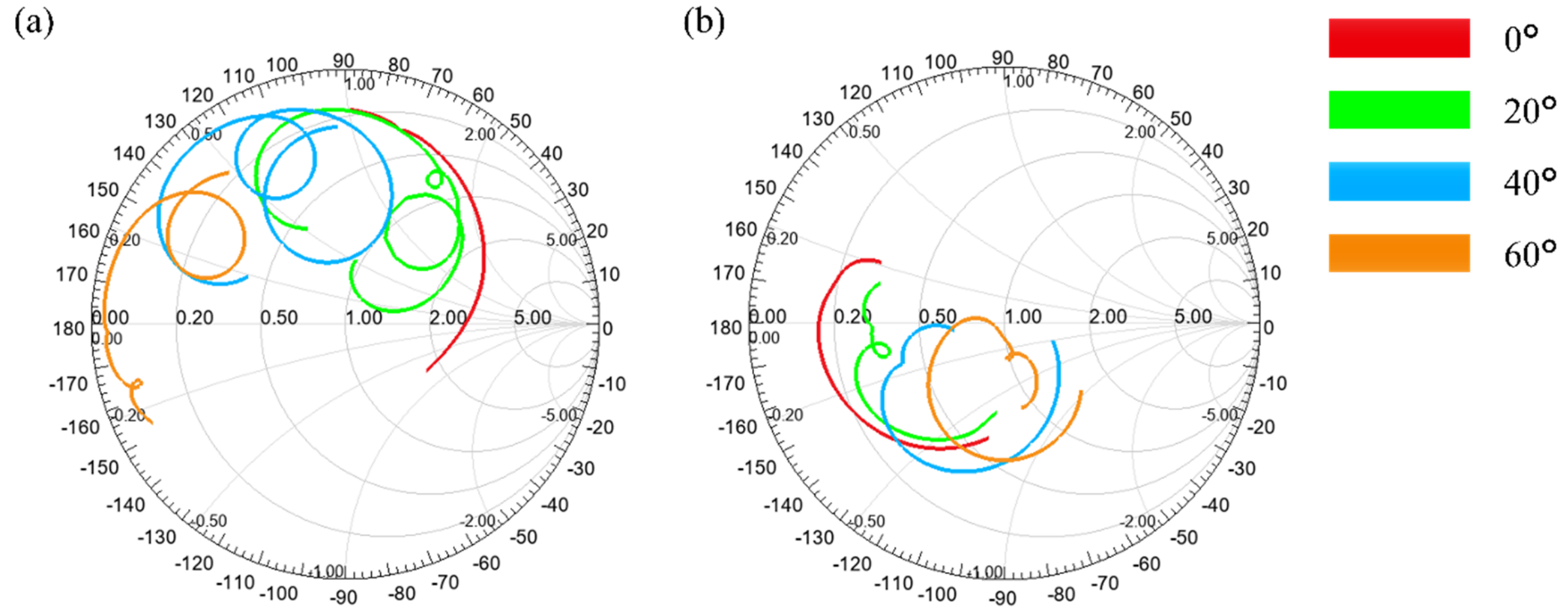

Fig. S3. Simulated Smith chart results of the unit cell for different incident angles: (a) OFF state and (b) ON state.

Fig. S3 shows the simulated Smith charts of the unit cell in the OFF and ON states for different incident angles. As the incident angle increases, the impedance trajectory shifts significantly in both states. In the OFF state, the impedance generally shifts toward a lower-impedance region, whereas in the ON state, it shifts toward a higher-impedance region.

Supplementary Note 4. Configuration of DC Bias Pads and Lines

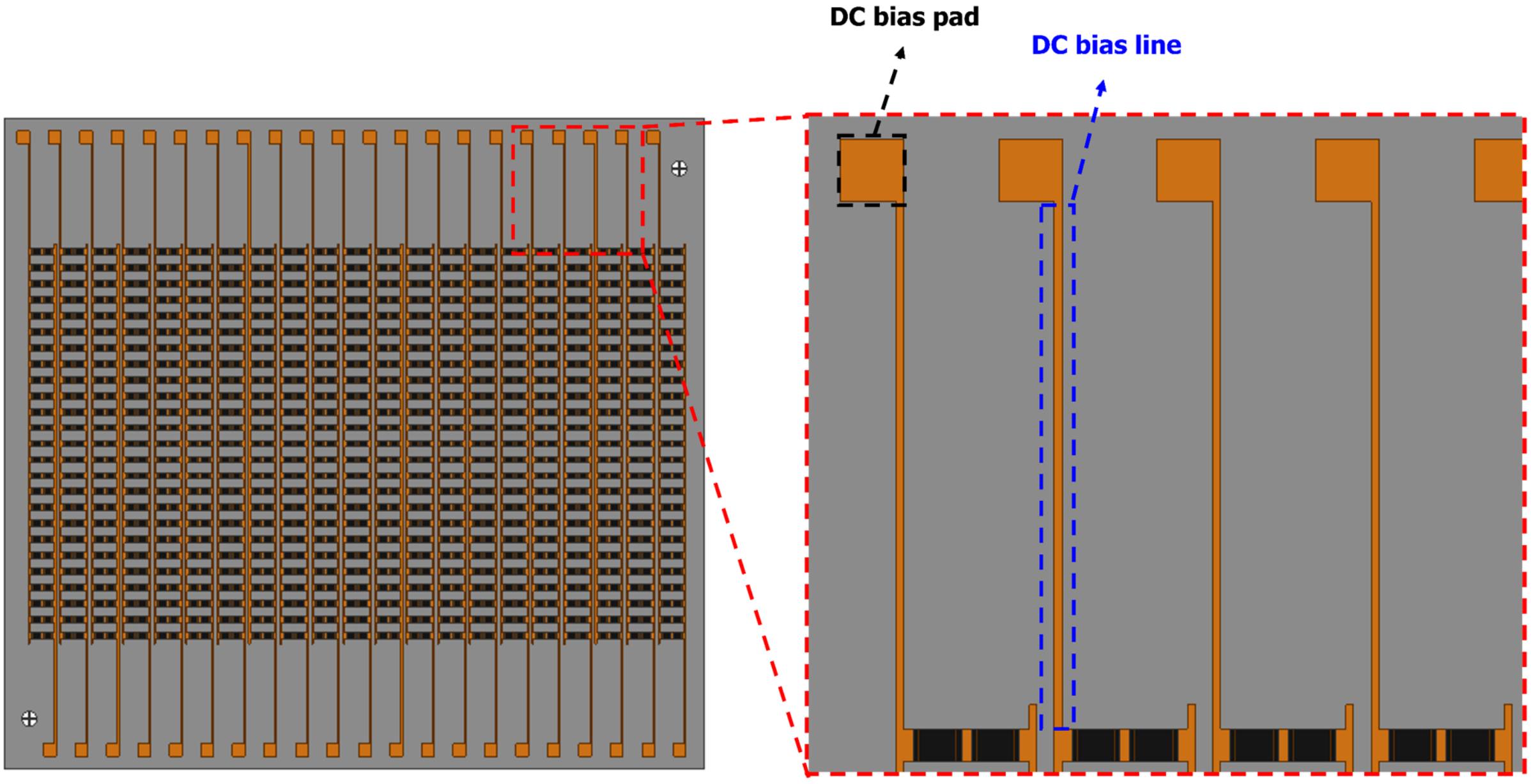

Fig. S4. Configuration of the proposed TRIS DC bias pads and bias lines

A DC biasing network was designed to control the VO_2 switches in the proposed TRIS. The TRIS is controlled on a column-by-column basis, and a parallel biasing scheme is implemented as shown in Fig. S4. The DC bias pads were designed to be 2.5 mm × 2.5 mm to accommodate jumper-wire soldering.

Supplementary Note 5. Alignment Using Screen-Printed Markers

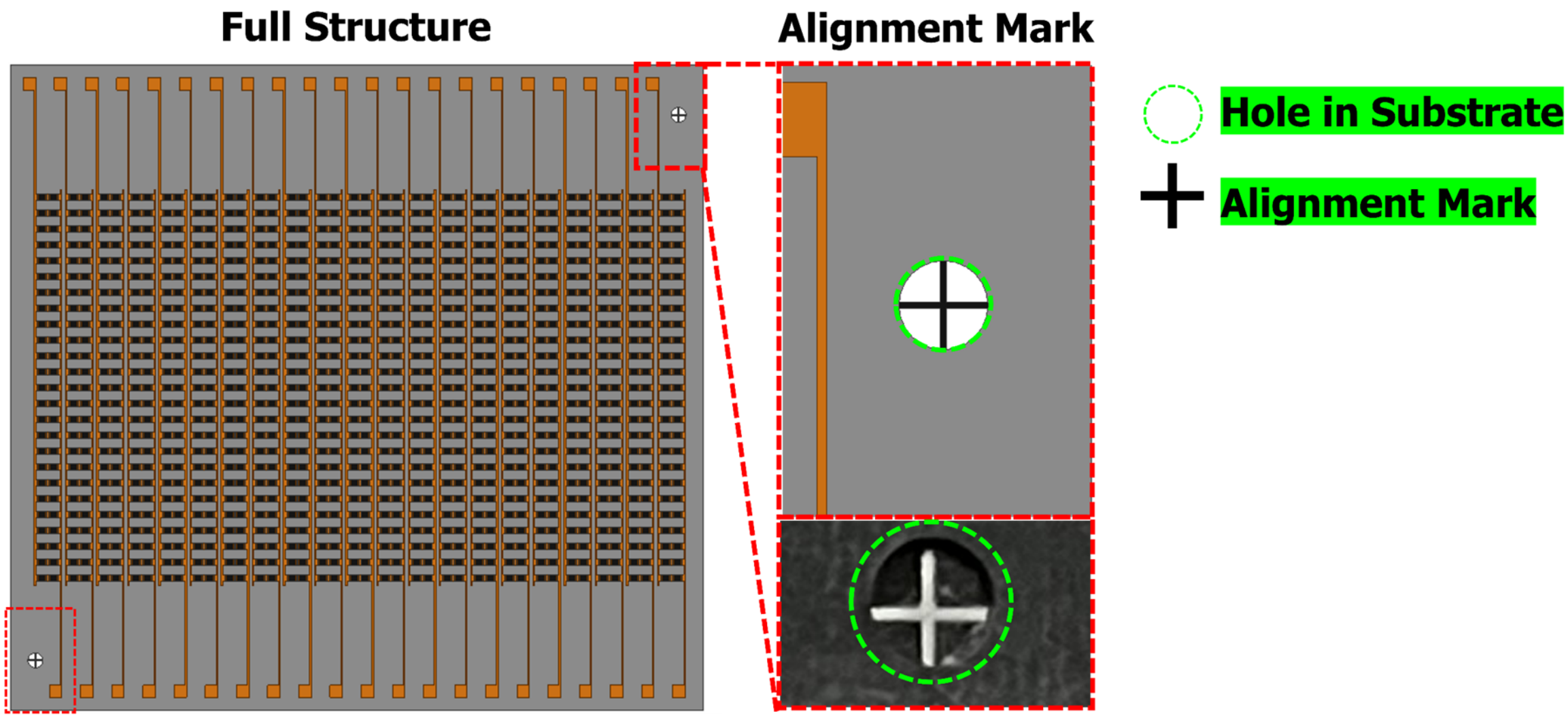

Fig. S5. Cross-shaped alignment marks and the corresponding alignment holes in the substrate.

Printing the VO_2 switches on both sides of the structure requires strict alignment. To satisfy this requirement, holes were drilled into the PCB substrate, and alignment marks were screen-printed to match these holes. During PCB fabrication, 3 mm diameter holes were created in the substrate. Corresponding cross-shaped alignment marks (0.2-mm line width, 3 mm × 3 mm overall size) were then printed on the screen-printing mask. During the screen-printing process, silver ink was first printed and dried on the working plate, after which the printed alignment marks were visually matched with the marks visible through the diagonally positioned hole. This ensured accurate double-sided alignment. Fig. S5 illustrates the designed hole-and-mark alignment scheme and the resulting alignment accuracy.

Supplementary Note 6. Effect of Air-Gap Height on TRIS Performance

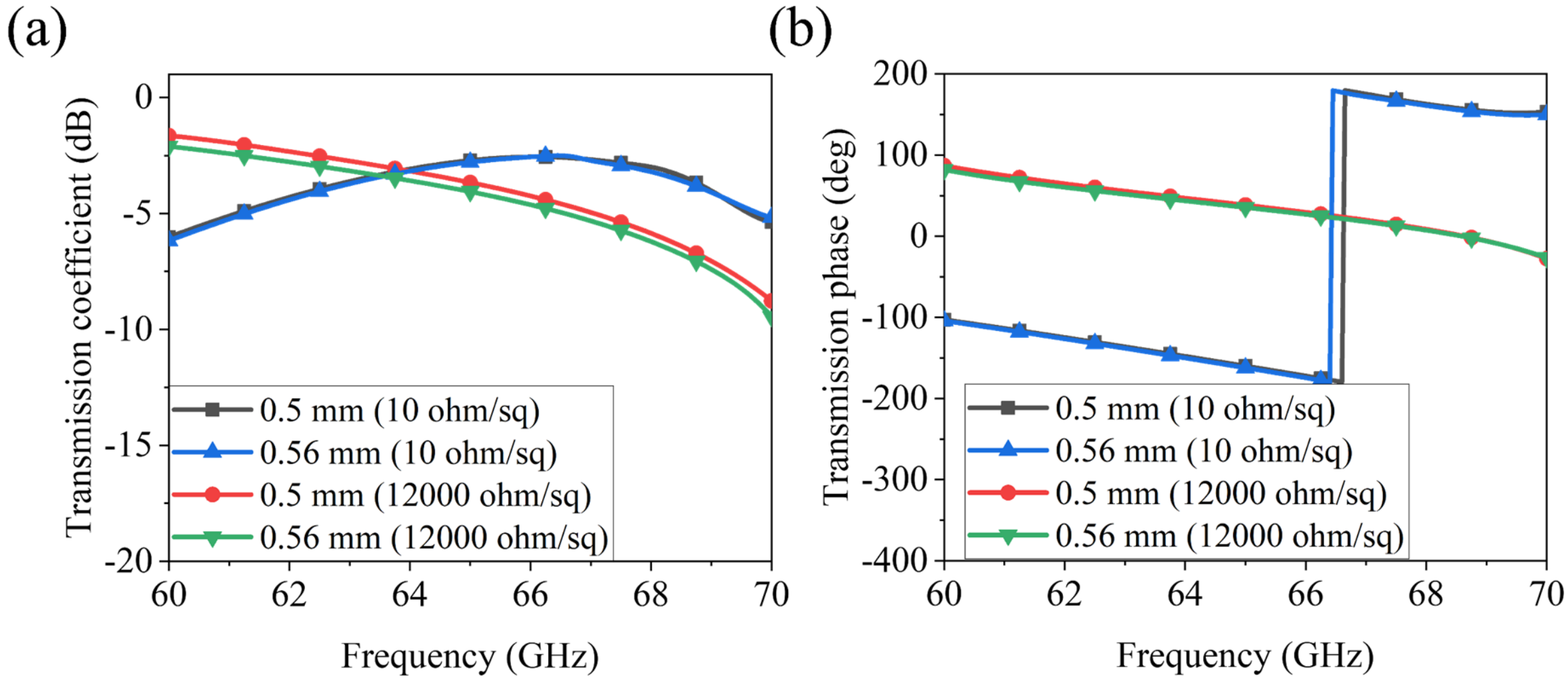

Fig. S6. Simulation results of (a) the transmission coefficient and (b) the transmission phase of the TRIS unit cell as a function of the air-gap height h_{air}.

Fig. S6 shows the simulated transmittance and phase results for TRIS unit cells with air gaps of 0.5 mm and 0.56 mm. Fig. S6(a) shows that the transmission coefficient loss in the OFF state is approximately 0.5 dB greater, while in the ON state, there was no difference between the two values. Overall, a similar trend can be observed in both states. Fig. S6(b) shows that there is no significant difference in the transmission phase between the OFF and ON states.

Supplementary Note 7. Full-Structure Simulation Setup

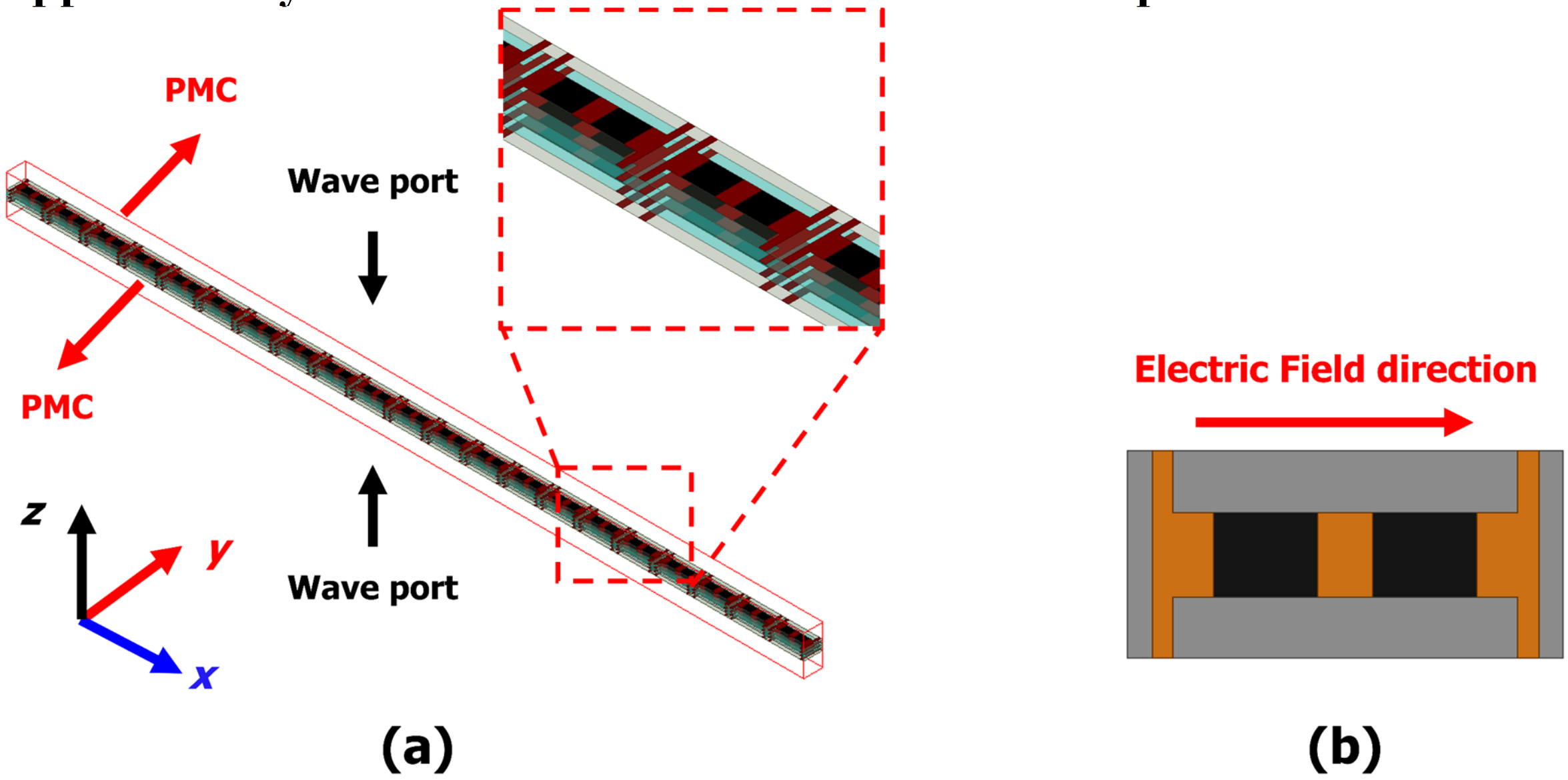

Fig. S7. (a) Full-wave simulation setup in Ansys HFSS for a 1-D metasurface composed of *N* unit cells, modeled using PMC boundaries and wave ports. (b) Electric field direction of the proposed TRIS unit-cell structure.

To verify the beam-scanning performance of the proposed TRIS, a full-structure simulation was conducted. Typically, such simulations are performed using an array identical to the manufactured structure. However, because of the multi-layer configuration of our design, the available computational resources were insufficient to simulate the full structure. Therefore, we adopted a propagation model for an *N* unit cell metasurface, as shown in Fig. S7(a). As illustrated in Fig. S7(b), the proposed TRIS is illuminated by an x-polarized uniform plane wave. To model the structure as being infinitely periodic along the y-axis, a perfect magnetic conductor (PMC) boundary was applied. This full-structure simulation approach has been validated in previous studies [1].

Supplementary Note 8. Comparison Between Analytical Calculation and EM Simulation

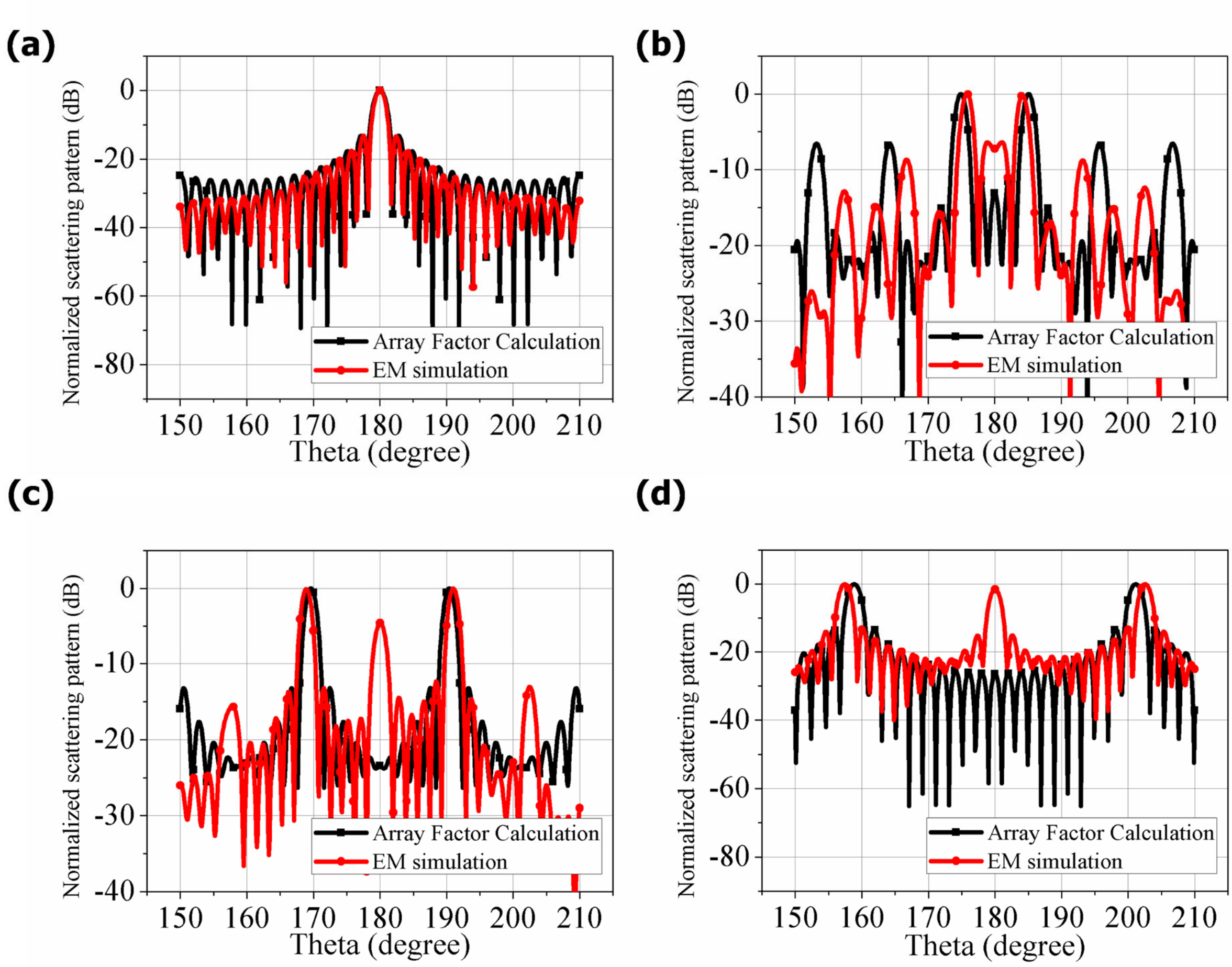

Fig. S8. Comparison of the simulation and calculation results at 62 GHz for (a) code 0000, (b) code 00001111, (c) code 0011, and (d) code 0101.

The theoretical calculation results and full-wave analysis results were compared to evaluate the beam-scanning angles of the overall proposed TRIS structure. The theoretical calculation results were obtained using the array-factor formula implemented in MATLAB. Figs. S8(a–d) present the normalized scattering patterns for each code, comparing the theoretical calculation results with the full-wave analysis results. Here, code 0 denotes columns with the VO_2 switch in the OFF state, and code 1 denotes columns with the VO_2 switch in the ON state. The beam-scanning angles for each code show close agreement, with the differences between the two results being within 1.4°. However, an additional intermediate beam appears in all codes except 0000, which occurs because the theoretical calculation does not account for coupling between adjacent unit cells.

Supplementary Note 9. Comparison Between Co- and Cross-polarization

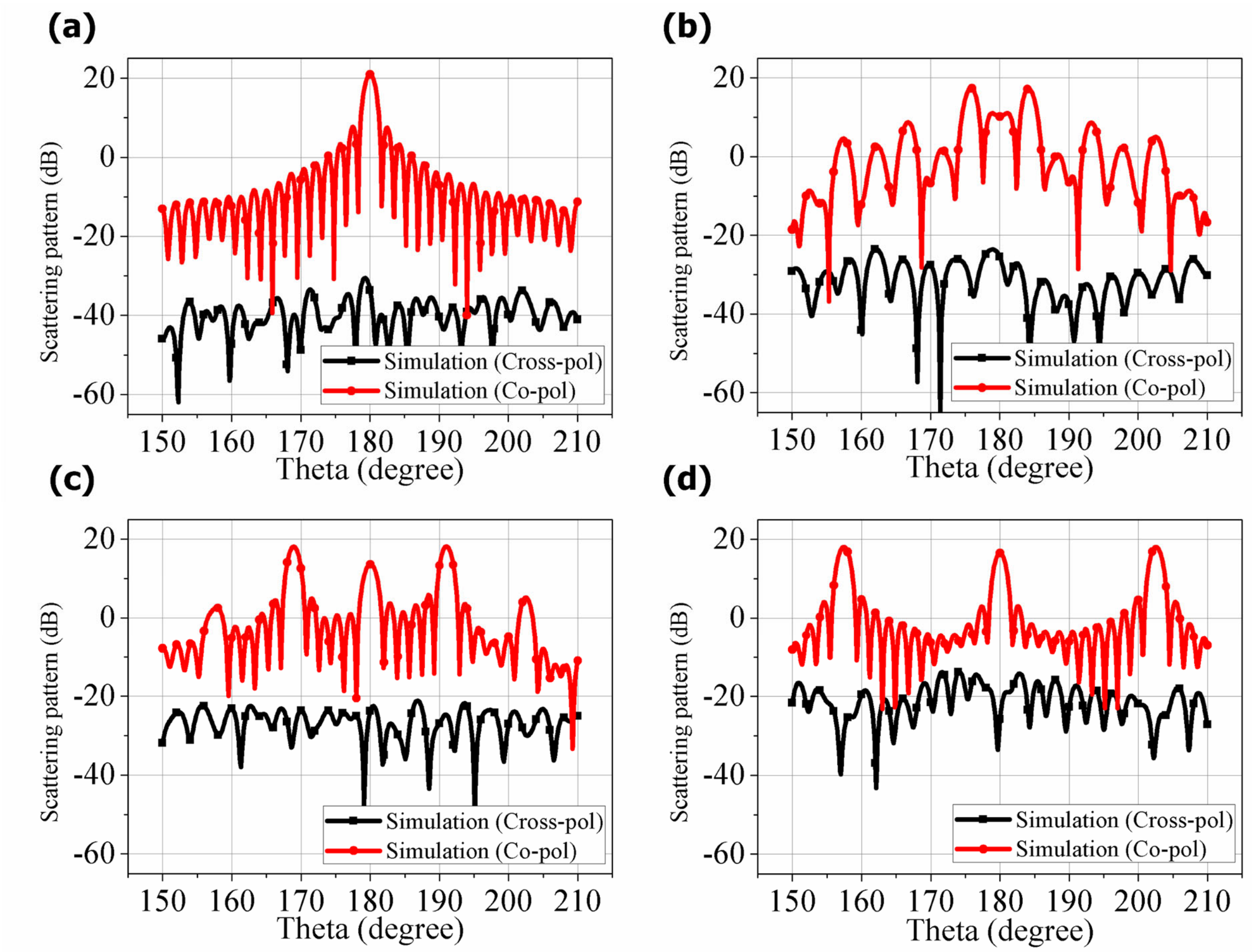

Fig. S9. Simulated co-polarized and cross-polarized scattering patterns at 62 GHz for (a) code 0000, (b) code 00001111, (c) code 0011, and (d) code 0101.

Fig. S9 presents the co-polarization and cross-polarization radiation patterns obtained from the full-structure simulations for each coding configuration. For code 0000, the maximum co-polarized intensity is 20.9 dB (Fig. S9(a)), while code 00001111 yields a maximum intensity of 17.23 dB (Fig. S9(b)). Code 0011 exhibits a maximum intensity of 18.19 dB (Fig. S9(c)), and code 0101 produces a maximum intensity of 18.07 dB (Fig. S9(d)). Except for code 0000, all other codes show more than a 3 dB reduction in maximum intensity compared to code 0000. This reduction occurs because the 1-bit quantization error causes the transmitted wave to split into two beams, dividing the available power. Additionally, for all codes, the cross-polarized components remain more than 40 dB lower than the corresponding co-polarized peak intensities.

Supplementary Note 10. Temperature Response of the 21-Column VO_2 Switch Array

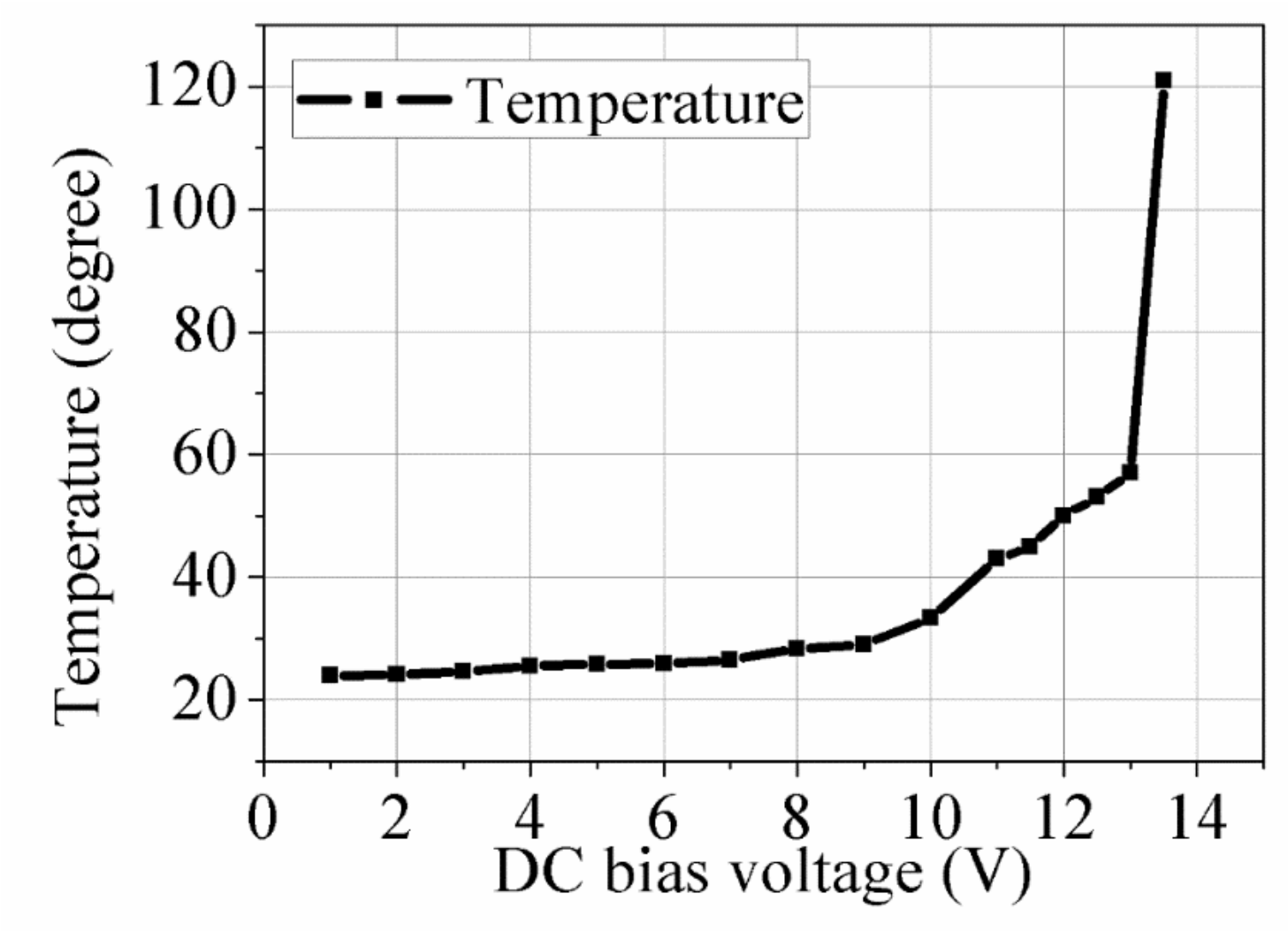

Fig. S10. Temperature variation as the applied DC voltage to the 21 parallel-connected columns changes.

Fig. S10 shows the temperature variation of the VO_2 switch as the applied DC bias voltage increases. The temperature rises gradually at lower voltages and then exhibits a sharp increase when the voltage increases from 13 V to 13.5 V. This abrupt temperature jump occurs because the VO_2 undergoes a rapid insulator–metal phase transition, which causes a sudden decrease in its resistance and results in a corresponding spike in temperature.

Reference

[1] Demetre, J. S., Smy, T. J. & Gupta, S. Static metasurface reflectors with independent magnitude and phase control using coupled resonator configuration. *IEEE Transactions on Antennas and Propagation* **71**, 3536–3545 (2023). https://doi.org/10.1109/TAP.2023.3242076
